# Supplementary material for: Randomised controlled feasibility trial of real versus sham repetitive transcranial magnetic stimulation treatment in adults with severe and enduring anorexia nervosa: the TIARA study
Source: BMJ Open. 2018 Jul 16;8(7):e021531. doi: 10.1136/bmjopen-2018-021531 (PMC6082449; doi:10.1136/bmjopen-2018-021531)
Supplement: Supplementary file 1 [file bmjopen-2018-021531supp001.pdf]

SUPPLEMENTARY METHODS

Blinding

Allocation concealment:

Unblinded treatment arm allocations were sent to a member of research staff who was independent from the trial team and had no contact with participants. Prior to trial commencement, this researcher labelled the real and sham rTMS coils with a coloured (red/green) keyring to blind the rTMS therapists to treatment allocation. This researcher then converted the unblinded randomisation allocation into the blinded treatment codes (red/green), and passed on this blinded allocation information to the rTMS therapists.

Assessment of blinding success:

Following a participant's rTMS treatment (at approximately 1 month post-randomisation), all researchers involved in their treatment provided a guess as to the participant's treatment allocation, and indicated certainty using a 10cm visual analogue (VAS) scale.

Every 5 sessions, participants were asked to guess their treatment allocation and record their certainty in their guess using a 10cm VAS scale.

Safety, tolerability and participants' experience of treatment

Assessment of side-effects:

rTMS-associated side effects were assessed at the beginning of each session (except session 1), with participants reporting on a 10cm visual analogue scale (VAS) whether they had experienced any of the following common rTMS side effects: headache, discomfort on the head, nausea, dizziness, light headedness, skin irritation or drowsiness. Participants were also able to record any other side effects they experienced.

Participants' expectations and experience of treatment:

Semi-structured qualitative interviews assessing treatment expectations and experience were conducted at follow-up (4-months post-randomisation). Findings will be reported in full elsewhere. A VAS of participants' expectations regarding the effects of rTMS on their condition (0 = 'I expect to feel much worse' and 10 = 'I expect to feel much better') was carried out in their first rTMS session.

SUPPLEMENTARY RESULTS

Blinding

Participant blinding:

Profile plots of participants' blinding guess combined with their rating of certainty are presented below separately for the real (see Figure 1) and sham (see Figure 2) intervention group. Each line in the profile plot refers to a different participant, the thick black line provides the median. A data point in the top part of the graph reflects that the participant guessed real. A data point in the bottom part of the graph reflects that the participant guessed sham. Certainty scores are reflected on the scale of 0 - 10/-10, with 0 meaning that the participant was completely unsure of their blinding decision and 10/10 meaning the participant was completely sure of their blinding decision.

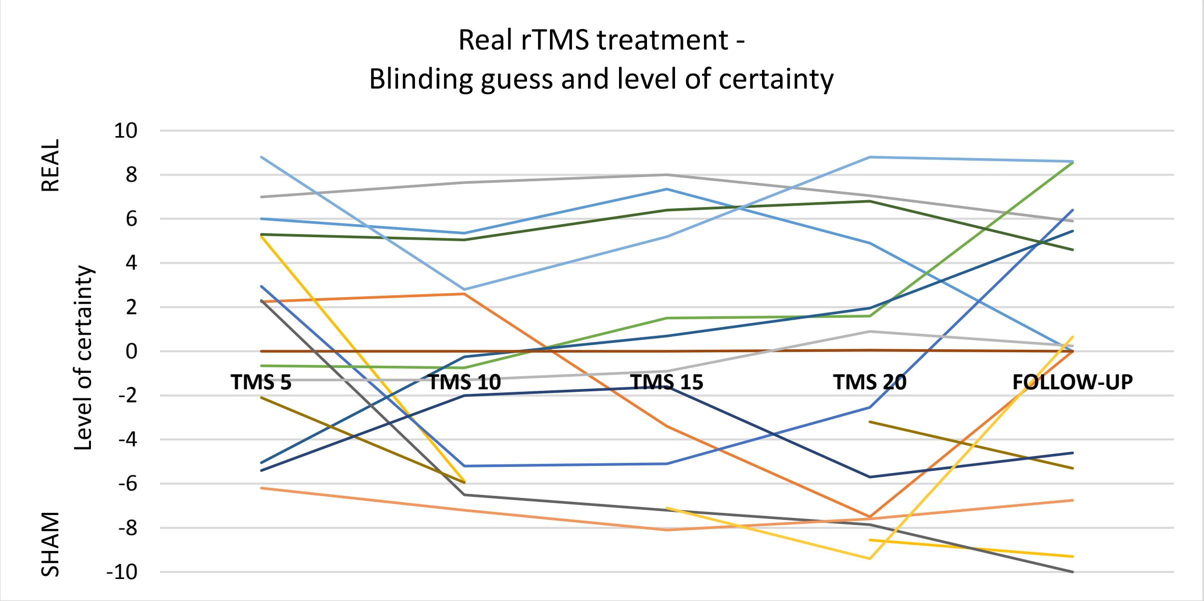

Figure 1. Blinding guesses combined with level of certainty for all participants in the real rTMS intervention group.

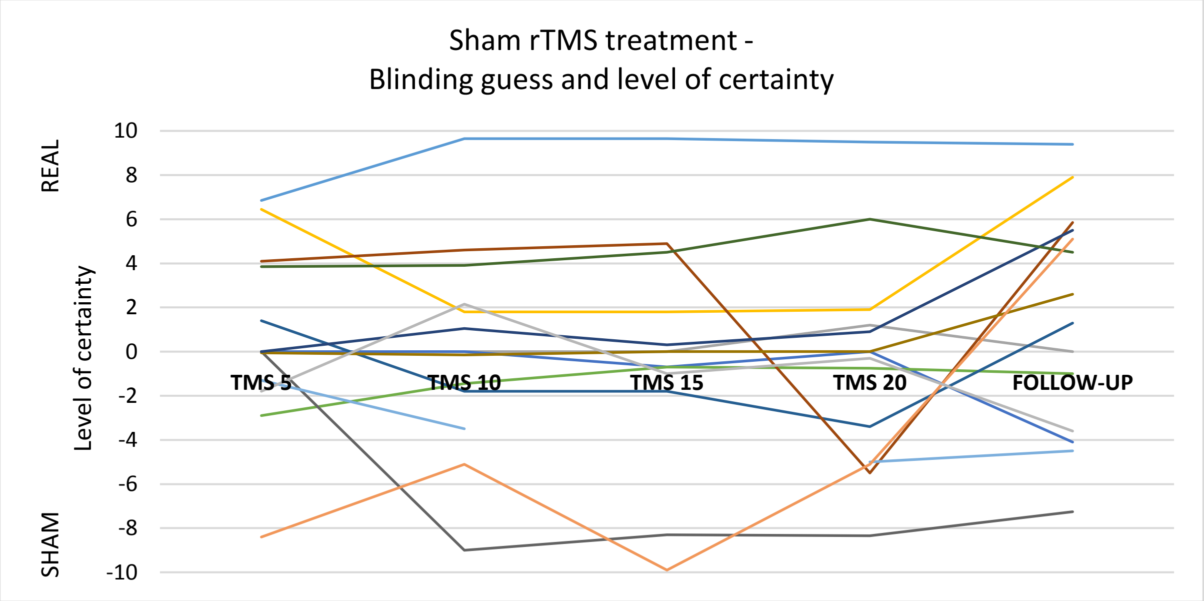

Figure 2. Blinding guesses combined with level of certainty for all participants in the sham rTMS intervention group.

Researcher blinding:

The rTMS therapists provided an allocation guess for each participant that they had administered rTMS to, once the participant had completed treatment. JM provided blinding guesses on n=17, correctly guessing the allocation of 41% of these participants. SB provided blinding guesses on n=29, correctly guessing the allocation of 31% of these participants. MK providing blinding guesses on

n=26, correctly guessing the allocation of 100% of these participants. Generally, certainty in their blinding guesses increased as the trial progressed.

Safety, tolerability, side effects and participants' expectations and experience of treatment

Tolerability and side effects:
Mean frequency and maximum intensity/severity of reported side effects are presented in Supplementary Table 1. In both groups, headaches were the most frequently reported side effect, followed by drowsiness in the real group and nausea in the sham group. Generally, the intensity/severity of side effects reduced as rTMS sessions progressed. Mean frequency of side effects was slightly higher in the real group compared to the sham group; however, the sham group experienced the same range of side effects and some had greater reported intensity/severity (e.g., headaches, nausea, dizziness, light-headedness). One participant reported chest pains as a side effect. Sessions were postponed and resumed after two days once the participant was medically cleared.

Treatment expectations:
The mean rating of participants' expectation of the efficacy of rTMS sessions in improving their condition was 6.63 (SD 1.78, range 1.8-10), with the majority of participants (n=27) expecting some clinical improvement (rating of 5-10).

Treatment experience:
Preliminary analysis of the qualitative interviews (full analyses of these data will be presented elsewhere; NB 29 transcripts available, *n*=1 transcript missing due to technical problems with recording) of patient treatment experiences suggests that many participants found attending the rTMS clinic a "very positive" and "really interesting" experience and they "enjoyed the sessions" (22/29 participants). Several participants (14/29) highlighted practical difficulties and the commitment involved: "it was quite hard travelling up every day", "it would be easier if you didn't have to do it every day", "took a lot of time and commitment"; however, some found it "much easier" than they thought it would be once they "got used to the routine" (7/29). Participants (18/29) also reported that whilst rTMS initially felt uncomfortable due to the tapping sensation on the head, any discomfort experienced lessened over time as they "got used to it".

	Real					Sham				
**Side effects**	*Total*	Sessions 2-5	Sessions 6-10	Sessions 11-15	Sessions 16-20	*Total*	Sessions 2-5	Sessions 6-10	Sessions 11-15	Sessions 16-20
Frequency of experience side effects(Mean (SD))										
Headache	13.25 (16.08)	2.75 (1.44)	3.88 (1.41)	3.50 (1.97)	3.13 (1.78)	8.25 (6.96)	2.56 (1.50)	2.50 (2.25)	1.69 (2.02)	1.50 (2.00)
Discomfort on head	9.19 (8.07)	2.00 (1.55)	2.50 (2.31)	2.56 (2.39)	2.13 (2.22)	5.44 (7.24)	2.00 (1.71)	1.44 (2.22)	1.06 (1.98)	0.94 (2.02)
Nausea	8.19 (7.48)	1.75 (1.65)	2.31 (2.33)	2.31 (2.12)	1.81 (1.94)	7.31 (7.65)	2.19 (7.65)	2.06 (1.38)	1.63 (2.36)	1.44 (2.25)
Dizziness	7.44 (7.62)	1.63 (1.75)	2.13 (2.31)	1.88 (2.06)	1.81 (2.07)	5.50 (7.13)	1.75 (1.73)	1.56 (2.10)	1.25 (1.95)	0.94 (2.02)
Light-headedness	8.13 (7.54)	1.81 (1.72)	2.13 (2.19)	2.31 (2.18)	1.88 (2.06)	6.69 (7.42)	2.00 (1.71)	1.88 (2.25)	1.50 (2.00)	1.31 (2.02)
Skin irritation	5.75 (6.82)	1.25 (1.39)	1.31 (1.74)	1.63 (2.16)	1.56 (1.97)	4.63 (7.40)	1.25 (1.73)	1.50 (2.31)	0.96 (1.88)	0.94 (2.02)
Drowsiness	10.38 (7.56)	2.25 (1.57)	3.00 (2.16)	2.88 (2.22)	2.25 (2.18)	7.06 (7.09)	1.88 (1.71)	2.38 (2.13)	1.38 (2.09)	1.44 (2.03)
Maximum intensity / severity of experienced side effects (Mean (SD))										
Headache	4.93 (2.29)	3.01 (2.69)	3.86 (2.33)	2.04 (2.06)	1.99 (2.42)	5.16 (2.77)	4.7 (3.05)	3.22 (3.00)	1.52 (1.83)	1.16 (1.84)
Discomfort on head	3.22 (3.26)	2.78 (3.11)	1.95 (3.07)	0.73 (0.96)	0.77 (1.62)	2.93 (3.14)	3.05 (3.21)	0.69 (1.80)	0.13 (0.29)	0.05 (0.16)
Nausea	2.91 (3.18)	2.34 (2.81)	1.67 (2.91)	0.58 (0.79)	0.91 (2.11)	3.46 (3.11)	2.42 (2.73)	1.34 (2.70)	1.46 (2.81)	1.09 (2.31)
Dizziness	1.70 (2.16)	1.08 (1.98)	0.99 (1.92)	0.56 (1.63)	0.91 (1.86)	3.29 (2.96)	2.05 (3.04)	1.05 (1.63)	1.31 (1.98)	0.19 (0.49)
Light-headedness	2.33 (2.60)	1.53 (2.15)	1.54 (2.44)	1.11 (2.09)	0.73 (1.73)	2.95 (3.01)	2.13 (2.97)	1.29 (2.18)	1.76 (2.69)	0.42 (0.75)
Skin irritation	0.81 (1.15)	0.39 (0.59)	0.45 (0.83)	0.34 (0.89)	0.33 (0.89)	0.68 (2.05)	0.44 (1.25)	0.67 (2.13)	0.19 (0.57)	0.05 (0.13)
Drowsiness	5.23 (3.60)	4.06 (3.41)	3.94 (3.62)	2.38 (2.80)	2.05 (2.58)	3.51 (3.16)	2.94 (3.28)	2.89 (2.74)	1.27 (2.59)	1.26 (2.14)

Supplementary Table 1. Mean frequency and mean maximum intensity / severity of experienced side effects.

SD = standard deviation
