# Supplementary material for: Randomised controlled feasibility trial of real versus sham repetitive transcranial magnetic stimulation treatment in adults with severe and enduring anorexia nervosa: the TIARA study
Source: BMJ Open. 2018 Jul 16;8(7):e021531. doi: 10.1136/bmjopen-2018-021531 (PMC6082449; doi:10.1136/bmjopen-2018-021531)
Supplement: Author's manuscript [file bmjopen-2018-021531.draft_revisions.pdf]

BMJ Open is committed to open peer review. As part of this commitment we make the peer review history of every article we publish publicly available.

When an article is published we post the peer reviewers' comments and the authors' responses online. We also post the versions of the paper that were used during peer review. These are the versions that the peer review comments apply to.

The versions of the paper that follow are the versions that were submitted during the peer review process. They are not the versions of record or the final published versions. They should not be cited or distributed as the published version of this manuscript.

BMJ Open is an open access journal and the full, final, typeset and author-corrected version of record of the manuscript is available on our site with no access controls, subscription charges or pay-per-view fees (http://bmjopen.bmj.com).

If you have any questions on BMJ Open's open peer review process please email info.bmjopen@bmj.com

BMJ Open

A randomised controlled feasibility trial of real versus sham repetitive transcranial magnetic stimulation treatment in adults with severe and enduring anorexia nervosa: the TIARA study.

Journal:	*BMJ Open*
Manuscript ID	bmjopen-2018-021531
Article Type:	Research
Date Submitted by the Author:	05-Jan-2018
Complete List of Authors:	Dalton, Bethan; Institute of Psychiatry, Psychology & Neuroscience, King's College London, Section of Eating Disorders, Department of Psychological Medicine<br>Bartholdy, Savani; Institute of Psychiatry, Psychology & Neuroscience, King's College London, Section of Eating Disorders, Department of Psychological Medicine<br>McClelland, Jessica; Institute of Psychiatry, Psychology & Neuroscience, King's College London, Section of Eating Disorders, Department of Psychological Medicine<br>Kekic, Maria; Institute of Psychiatry, Psychology & Neuroscience, King's College London, Section of Eating Disorders, Department of Psychological Medicine<br>Rennalls, Samantha; Institute of Psychiatry, Psychology & Neuroscience, King's College London, Section of Eating Disorders, Department of Psychological Medicine<br>Werthmann, Jessica; Institute of Psychiatry, Psychology & Neuroscience, King's College London, Section of Eating Disorders, Department of Psychological Medicine<br>Carter, B; Institute of Psychiatry, Psychology & Neuroscience, King's College London, Department of Biostatistics and Health Informatics<br>O'Daly, Owen; Institute of Psychiatry, Psychology & Neuroscience, King's College London, Centre for Neuroimaging Sciences, Department of Neuroimaging<br>Campbell, Iain; Institute of Psychiatry, Psychology & Neuroscience, King's College London, Section of Eating Disorders, Department of Psychological Medicine<br>David, Anthony; Institute of Psychiatry, Psychology & Neuroscience, King's College London, Department of Psychosis Studies<br>Glennon, Danielle; South London and Maudsley NHS Foundation Trust<br>Kern, Nikola; South London and Maudsley NHS Foundation Trust<br>Schmidt, Ulrike; Institute of Psychiatry, Psychology & Neuroscience, King's College London, Section of Eating Disorders, Department of Psychological Medicine
Keywords:	Eating disorders < PSYCHIATRY, Neuromodulation, Repetitive transcranial magnetic stimulation (rTMS), Anorexia nervosa

SCHOLARONE™
Manuscripts

For peer review only

A randomised controlled feasibility trial of real versus sham repetitive transcranial magnetic stimulation treatment in adults with severe and enduring anorexia nervosa: the TIARA study.

Bethan Dalton[1]*, Savani Bartholdy[1]*, Jessica McClelland[1]*, Maria Kekic[1], Samantha J. Rennalls[2], Jessica Werthmann[1], Ben Carter[3], Owen G. O'Daly[2], Iain C. Campbell[1], Anthony S. David[4], Danielle Glennon[5], Nikola Kern[5] and Ulrike Schmidt[1,5#]

1. Section of Eating Disorders, Department of Psychological Medicine, Institute of Psychiatry, Psychology & Neuroscience, King's College London, London, UK

2. Centre for Neuroimaging Sciences, Department of Neuroimaging, Institute of Psychiatry, Psychology & Neuroscience, King's College London, London, UK

3. Department of Biostatistics and Health Informatics, Institute of Psychiatry, Psychology & Neuroscience, King's College London, London, UK

4. Department of Psychosis Studies, Institute of Psychiatry, Psychology & Neuroscience, King's College London, London, UK.

5. South London and Maudsley NHS Foundation Trust, London, UK

*Authors contributed equally

# Corresponding author: Professor Ulrike Schmidt, PO59 Section of Eating Disorders, Department of Psychological Medicine, Institute of Psychiatry, Psychology and Neuroscience, King's College London, London, SE5 8AF, UK. ulrike.schmidt@kcl.ac.uk.

Word count: 3998

For peer review only - http://bmjopen.bmj.com/site/about/guidelines.xhtml

ABSTRACT

Objective: Treatment options for severe, enduring anorexia nervosa (SE-AN) are limited. Non-invasive neuromodulation is a promising emerging intervention. Our study is a feasibility randomised controlled trial (RCT) of repetitive transcranial magnetic stimulation (rTMS) in individuals with SE-AN, which aims to inform the design of a future large-scale trial.

Design: Double-blind, parallel group, two-arm, sham-controlled trial.

Setting: Specialist eating disorders centre.

Participants: Community-dwelling people with AN, an illness duration of ≥3 years and at least one previous completed treatment.

Interventions: Participants received 20 sessions (administered over 4 weeks) of MRI-guided real or sham high-frequency rTMS to the left dorsolateral prefrontal cortex in addition to treatment-as-usual (TAU).

Outcomes: Recruitment, attendance and retention rates, clinical outcomes (body mass index [BMI], eating disorder symptoms, mood, quality of life), rTMS safety and tolerability. Assessments were conducted at baseline, post-treatment and follow-up (i.e., at 0-, 1- and 4-months post-randomisation).

Results: Thirty-four participants (17 per group) were randomly allocated to real or sham rTMS. One participant per group was withdrawn prior to the intervention due to safety concerns. Two participants (both receiving sham) did not complete the treatment. rTMS was safe and well tolerated. Between-group effect sizes of change scores (baseline to follow-up) were small for BMI (d=0.2, 95% CI -0.49-0.90) and eating disorder symptoms (d=0.1, 95% CI -0.60-0.79), medium for quality of life and moderate to large (d=0.61 to 1.0) for mood outcomes, all favouring rTMS over sham.

Conclusions: The treatment protocol is feasible and acceptable to participants. Outcomes provide preliminary evidence for the therapeutic potential of rTMS in SE-AN. Largest effects were observed on variables assessing mood. This study supports the need for a larger confirmatory trial to evaluate the effectiveness of multi-session rTMS in SE-AN. Future studies should include a longer follow-up period and an assessment of cost-effectiveness.

Keywords

Eating disorders; neuromodulation; anorexia nervosa; repetitive transcranial magnetic stimulation (rTMS)

Strengths and limitations of this study

- This is the first randomised controlled feasibility trial of multi-session repetitive transcranial magnetic stimulation (rTMS) treatment people with anorexia nervosa (AN).
- It targeted those with severe and enduring AN (SE-AN).
- A range of outcomes were assessed (recruitment, retention, safety, tolerability and effect sizes of clinical outcome variables) and thus it provides useful data for implementing a larger scale randomised controlled trial of rTMS in SE-AN.
- The study had a small sample size, was not powered or designed to assess the efficacy of rTMS in SE-AN and the follow-up duration was short.

For peer review only - http://bmjopen.bmj.com/site/about/guidelines.xhtml

INTRODUCTION

Anorexia nervosa (AN) is a life-threatening brain-based disorder of multi-factorial aetiology. Alterations in neural circuits involved in reward processing, negative affect and stress, appetite regulation, cognitive (self-regulatory) control and socio-emotional processes have been implicated in its causation and maintenance [1, 2].

Approximately 20% of patients develop a severe, enduring form of illness (SE-AN) [3]. These patients typically have high levels of depression and anxiety, are socially isolated and markedly impaired in their ability to care for themselves. In fact, their quality of life is comparable to that of patients with depression and impairments in social contact and self-care are comparable to those in psychosis [4]. There has been significant growth in research on treatments for AN, with much of this focusing on psychological therapies [5]. Only two small trials have focused on SE-AN, using psychological therapies or medication to improve quality of life [6] or achieve weight gain, albeit with limited success [7]. Thus, treatment options for these patients are limited [6, 8, 5, 1] and there is a need for novel interventions for this group.

Non-invasive neuromodulation is a promising emerging treatment for SE-AN [5, 9, 2], in particular repetitive transcranial magnetic stimulation (rTMS) [e.g., 10, 11]. rTMS can enhance (high-frequency) or inhibit (low-frequency) cortical activity in targeted brain areas. It appears to increase neuroplasticity, and hence may be of value in chronic or treatment-resistant neurocircuit-based disorders, such as SE-AN [9]. Based on the Research Domain Criteria, candidate targets for rTMS in eating disorders (EDs) have been described, involving brain structures/circuitry in the cognitive control, positive and negative valences, and social processes systems [12]. Partly for theoretical reasons, but also for pragmatic accessibility reasons, rTMS studies have targeted the dorsolateral prefrontal cortex (DLPFC) or the dorsomedial prefrontal cortex (DMPFC) [12].

Proof-of-concept studies have shown that rTMS is a promising treatment in AN [9, 12]. We previously carried out two single-session studies in AN [13, 14] and a small case series of 20 sessions of rTMS in SE-AN [15, 11], all involving high-frequency rTMS to the left DLPFC. These studies showed that rTMS can lead to both short- and long-term improvements in ED symptoms, mood and reward-based decision making. Thus, there is a rationale for further exploring the therapeutic potential of rTMS in SE-AN.

To date, no sham-controlled RCT of rTMS in SE-AN has been conducted. The present trial (TIARA, Transcranial Magnetic Stimulation and Neuroimaging in Anorexia Nervosa) aimed to assess the feasibility of using rTMS compared to sham treatment in people with SE-AN and to inform the development of a large-scale sham-controlled RCT [16]. Our primary objective was:

a) To assess recruitment, attendance and retention rates.

Secondary objectives were:

a. To estimate the treatment effect sizes and standard deviations for outcome measures to inform future sample size calculations.
b. To determine safety and tolerability of rTMS in SE-AN.

Subsidiary objectives were to assess neuropsychological and neural correlates and predictors of rTMS treatment in AN, and to assess within-session change processes. Findings relating to these will be published elsewhere. The study aims, trial design and methodology are described fully in a protocol paper [16].

For peer review only - http://bmjopen.bmj.com/site/about/guidelines.xhtml

METHODS

Design, participants and setting

In a double-blind parallel group, randomised control design, participants were allocated to receive 20 sessions of either real or sham high-frequency rTMS in addition to treatment-as-usual (TAU). Outcomes were assessed at baseline (pre-randomisation), post-treatment (~ 1-month post-randomisation) and at follow-up (~ 4-months post-randomisation).

Right-handed adults (≥18 years old) with a current Diagnostic and Statistical Manual of Mental Disorders (5th Edition [17]; DSM-5) diagnosis of AN and a BMI >14 kg/m^2 were eligible. Participants had to have a severe, enduring form of the disorder; this was defined as an illness duration of ≥3 years and completion of at least one previous course of treatment (e.g., National Institute for Health and Care Excellence ([18]; NICE)-recommended specialist psychotherapy or specialist day-care or inpatient treatment for their ED). To take part, participants needed agreement from their ED clinician or general practitioner. Main exclusion criteria were related to contraindications to either rTMS or magnetic resonance imaging (MRI) [for details, see 16].

Participants were recruited from the Eating Disorders Unit at the South London and Maudsley NHS Foundation Trust, through online and media advertisements and through participation in other research projects. Ethical approval was given by the London - City Road & Hampstead Research Ethics Committee (REC ref: 15/LO/0196).

Potential participants underwent a screening procedure to determine eligibility [see 16 for details]. Once eligibility was determined, participants' written informed consent was obtained prior to the baseline assessment.

Randomisation and blinding

Randomisation was conducted by the King's College London (KCL) Clinical Trials Unit (CTU) using their automatic online system. Randomisation requests were submitted by study researchers via the web-based CTU system upon completion of the baseline assessment. Participants were allocated at a ratio of 1:1 to the two trial arms using a restricted stratified randomisation algorithm stratifying by prognostic factors: AN subtype (AN-restrictive or AN-binge-purge) and intensity of TAU (high: day care treatment, or low: outpatient treatment or no treatment). The stratification was implemented by minimised randomisation with a random component. The first n cases (n was not disclosed) were allocated entirely at random to further enhance allocation concealment.

Participants and researchers were blinded to treatment allocation, except for one researcher who conducted follow-up assessments and unblinded participants. For practical reasons, a small proportion of rTMS sessions (116/594 sessions; 19.53%) was delivered by the unblinded researcher. All other rTMS therapists remained blinded until study data had been collected and analysed. Participants were unblinded at 4-months post-randomisation once they had completed the study. Participants who received the sham intervention were offered real rTMS (if they continued to meet eligibility criteria) after their follow-up. Assessments of blinding success were carried out for rTMS therapists and participants. For details, see supplementary information.

Intervention

In both groups, participants received 20 sessions of (real or sham) high-frequency rTMS to the left DLPFC over 20 consecutive weekdays, in addition to TAU (i.e., specialist ED outpatient or day-care treatment, or no current treatment). Each session lasted 30 to 60 minutes, including preparation time, 20 minutes of rTMS and administration of within-session measures. rTMS sessions were conducted at the Institute of Psychiatry, Psychology & Neuroscience, KCL in a designated rTMS suite.

Prior to starting treatment, all participants underwent a structural MRI scan to localise the DLPFC (Talairach co-ordinates x = −45 y =45 z = 30) [19, 11] for the purpose of neuronavigation (using

For peer review only - http://bmjopen.bmj.com/site/about/guidelines.xhtml

Brainsight™ neuronavigation software). To determine the intensity of the rTMS stimulation, a Magstim Rapid device (Magstim®, Whitland, Wales, UK) with a real TMS figure-of-eight coil was used to determine participants' motor threshold (MT), which represents membrane-related excitability of cortical axons. Using the motor-evoked potential method, the MT was established by determining the minimum stimulator output intensity required to obtain five out of ten motor-evoked potentials >50 μV. MT was acquired weekly for each participant to ensure accuracy of the rTMS dose.

The Magstim Rapid device and Magstim D70-mm air-cooled real and sham coils were used to administer real and sham rTMS. Participants in the real group received 20 sessions of high-frequency (10 Hz) rTMS at 110 % of their individual MT, consisting of twenty 5-second trains with 55-second inter-train intervals delivered to the left DLPFC (a total of 1000 pulses delivered over each 20 minute session) [11, 19]. Sham stimulation was administered at the same parameters as real rTMS; however, a sham coil was used. The sham coil produces the same noises and feelings as the real coil but does not deliver active stimulation to the brain; rather it stimulates facial and scalp nerves.

Outcomes

The primary outcomes to assess feasibility were recruitment, attendance and retention rates. To judge whether or how to proceed with a future definitive trial we pre-specified two criteria, firstly, recruitment as planned (see protocol paper [16] and the 'Changes to planned protocol' section below) and, secondly, research follow-up rates of ≥ 80% at 4-months post-randomisation. We did not pre-specify any rTMS session attendance rates required for progression to a full trial, but clearly these would also guide a decision about the feasibility of a future trial. rTMS session attendance was recorded using a specially designed case record form.

Secondary feasibility outcomes included a range of clinical measures administered at baseline, 1-month (post-treatment) and 4-months post-randomisation (follow-up) to assess ED symptomatology, mood, other psychopathology and quality of life. Neurocognitive and neuroimaging assessments of rTMS treatment (see protocol paper [16]) were also completed, but will be presented elsewhere.

ED symptomatology: BMI; Eating Disorder Examination Questionnaire (EDE-Q) version 6.0 [20], Fear of Food Measure [21], Self-Starvation Scale (SS) [22], Eating Disorder Recovery Self-Efficacy Questionnaire (EDRSQ) [23].

Measures of mood and other psychopathology: Depression, Anxiety and Stress Scale - 21 item (DASS-21) [24], Positive and Negative Affect Schedule (PANAS) [25], Profile of Mood States (POMS) [26], Revised Obsessive-Compulsive Inventory (OCI-R) [27].

Quality of life: EuroQol Quality of Life Scale (EQ-5D-5L) [28], Clinical Impairment Assessment (CIA) [29, 30].

In light of the prominent mood and quality of life component of SE-AN, and the association between these two variables in SE-AN [4], the clinical outcome to be assessed as a primary outcome in a future definitive trial would most likely be the DASS.

Additional service utilisation: Patients' additional service utilisation was assessed with a self-report version of the Clinical Service Receipt Inventory (CSRI) [31] and a specially designed case record form.

Safety, tolerability and participants' experience of treatment:

To ensure safety, participants' weight, blood pressure (sitting and standing) and pulse were monitored weekly. Routine blood tests (including full blood count, urea and electrolytes, renal and liver function tests) were conducted prior to the start of rTMS treatment and were repeated at the mid-point of treatment or more frequently if clinically indicated. rTMS-associated side effects and participants' expectations and experience of treatment were also assessed (see supplementary files).

For peer review only - http://bmjopen.bmj.com/site/about/guidelines.xhtml

Procedure

Full details of the procedures and a table of measures-by-assessment are presented in our protocol paper [16]. All procedures were identical between groups, except for the rTMS intervention.

Baseline assessment: Participants' weight and height were measured and they completed a battery of questionnaires (described above) and neuropsychological computer tasks (not presented here). A one-hour MRI scan was also conducted. This included a structural MRI (for rTMS target localisation), functional MRI (fMRI), resting state fMRI and arterial spin labelling (not reported here). Thereafter, participants were randomly allocated to real or sham rTMS treatment.

All rTMS procedures and parameters were in accordance with the current safety and application guidelines for rTMS [32]. Treatment was delivered by researchers trained in rTMS administration.

Each rTMS session (except session 1) started with assessment of any side effects experienced since the previous session. Within-session ED cognitions were measured with VAS (relating to subsidiary aims, published separately), completed following brief cue exposure (i.e., film-clip of highly palatable foods) immediately before and after each rTMS session.

Post-treatment assessment (1-month post-randomisation): The post-treatment assessment occurred within one week of the final rTMS session and included the same elements as the baseline assessment.

Follow-up (4-months post-randomisation): This final assessment repeated the post-treatment assessment, except no MRI scan was conducted. In this session, an audio-recorded qualitative semi-structured interview was undertaken to ascertain participants' views on and experience of rTMS (published in full elsewhere) and blinding success was evaluated. Participants were then unblinded and individuals in the sham rTMS group were offered real rTMS treatment.

Changes to planned protocol

We planned to recruit 44 participants, but revised this to 30 participants because a greater than anticipated proportion of potential participants were not eligible (e.g., due to MRI/rTMS contraindications or being left-handed). These figures are in line with recommendations for feasibility trials [33] and accounted for attrition. Additionally, we removed the upper BMI limit (18.5 kg/m^2) to reflect the change in diagnostic criteria for AN in DSM-5 [17].

Data analysis

Primary feasibility outcomes are presented as n/N (%). The post-treatment and follow-up group means and standard deviations for secondary outcomes were adjusted for baseline and presented with effect sizes (Cohen's *d*) alongside 95% confidence intervals (CI). Last observation carried forward imputation was used for missing data.

RESULTS

Patient flow, attendance and retention

Patient flow is shown in the CONSORT diagram (Figure 1) and the primary feasibility outcome findings are described below. The trial duration was determined by the funding period.

During the 20-month recruitment period (August 2015-March 2017), 269 people expressed interest in the study. Thirty-four of these were enrolled and randomly allocated to the two treatment arms (n=17 per group). Two randomised participants were withdrawn for safety reasons prior to starting treatment: one participant (allocated to sham rTMS) had a syncope during her initial MT assessment; the other (allocated to real rTMS) was withdrawn as her weight had dropped below BMI 14 kg/m^2. These participants were excluded from the analyses. All others were included.

Thirty-two participants started treatment; two participants allocated to sham rTMS stopped treatment, one after 4 sessions (due to anxiety with travel) and one after 9 sessions (due to multiple

For peer review only - http://bmjopen.bmj.com/site/about/guidelines.xhtml

commitments). All other participants (n=30) completed treatment (defined a priori as ≥ 17 sessions of rTMS) and all three study assessments.

Baseline demographics and clinical characteristics are presented in Table 1. All participants were female and had a long-standing illness, having previously spent a mean of nearly 11 months as an inpatient for their ED.

Table 1. Baseline demographics and clinical characteristics

	**Whole sample**		**Real rTMS**		**Sham rTMS**	
	N		*N*		*N*	
**Demographic details**						
Age (mean [SD])	34	29.74 (10.35)	17	28.47 (9.48)	17	31.00 (11.29)
Highest level of education achieved (*n*)	33		17		16	
GCSE		3		2		1
AS Levels and above		30		15		15
Ethnicity (*n*)	34		17		17	
White		31		16		15
Other		3		1		2
Marital Status (*n*)	34		17		17	
Single		26		13		13
Married		6		4		2
Divorced		1		0		1
Other		1		0		1
**Clinical characteristics**						
Diagnosis (*n*)	34		17		17	
AN-R		22		11		11
AN-BP		12		6		6
BMI, kg/m^2 (mean [SD])	33	16.00 (1.44)	17	15.76 (1.62)	16	16.26 (1.22)
Duration of illness, years (mean [SD])	34	14.07 (10.75)	17	13.74 (10.74)	17	14.41 (11.09)
Number of previous ED hospitalisations (mean [SD])	34	2.18 (1.91)	17	2.47 (2.07)	17	1.88 (1.76)
Number of previous ED inpatient stays, months (mean [SD])	33	10.49 (11.66)	17	12.37 (12.46)	16	8.50 (10.78)
Current treatment (*n*)	34		17			
Receiving ED day care treatment		2		1		1
Receiving ED outpatient treatment		25		13		12
Receiving no treatment		7		3		4
Antidepressant medication		21		11		10
EDE-Q Global (mean [SD])	33	4.16 (1.11)	17	4.07 (1.28)	16	4.25 (0.94)
CIA Total (mean [SD])	33	43.64 (11.36)	17	43.35 (12.72)	16	43.94 (10.12)
EQ-5D-5L: How good or bad is your health today? (mean [SD])	33	48.91 (17.44)	17	47.47 (18.63)	16	50.44 (16.55)
DASS-21 Depression (mean [SD])	33	26.12 (9.68)	17	26.82 (9.44)	16	25.38 (10.19)
DASS-21 Anxiety (mean [SD])	33	15.39 (10.29)	17	14.82 (8.31)	16	16.00 (12.31)

For peer review only

DASS-21 Stress (mean [SD])	33	26.91 (7.92)	17	28.35 (7.12)	16	25.38 (8.66)
DASS-21 Total (mean [SD])	33	68.42 (24.52)	17	70.00 (20.59)	16	66.75 (28.72)
POMS Total Mood Disturbance (mean [SD])	33	83.97 (36.75)	17	81.41 (36.84)	16	86.69 (37.66)
OCI-R Total (mean [SD])	33	27.79 (16.97)	17	24.00 (16.48)	16	31.81 (17.05)

rTMS = repetitive transcranial magnetic stimulation; AN-R = anorexia nervosa restrictive subtype; AN-BP = anorexia nervosa binge-purge subtype; ED = eating disorder; EDE-Q = Eating Disorder Examination Questionnaire; DASS= Depression, Anxiety and Stress Scale; CIA = Clinical Impairment Assessment; EQ-5D-5L = EuroQol Quality of Life Scale; OCI-R = Revised Obsessive-Compulsive Inventory

Treatment effect sizes

The means, standard deviations and between-group treatment effect sizes (with confidence intervals) for change scores (baseline to post-treatment and baseline to follow-up) of the secondary clinical outcomes are presented in Table 2. Group differences in BMI and ED symptoms were of small effect at both post-treatment and follow-up, but favoured active treatment. At 4-months post-randomisation, there were between-group differences of medium to large effect size in measures of mood, obsessive compulsive symptoms and quality of life, all favouring the active treatment. The adjusted means for the planned future primary outcome, DASS total score, were -21.25 (SD 24.33) in the real intervention group and -3.75 (SD 12.75) in the sham group, with a between-group effect size of *d*= -0.9 (95% CI -1.62 to -0.17).

For peer review only

Table 2. The mean change scores (post-treatment and follow-up scores adjusted for baseline) for the secondary clinical outcome measures, including the number of participants included in the analysis (N), means, and standard deviations (SD) for each group, and the estimated effect size (Cohen's *d*) with 95% confidence intervals (95% CI).

	**Post-treatment (adjusted for baseline)**							**Follow-up (adjusted for baseline)**						
	**Real**			**Sham**			***d* (95% CI)**	**Real**			**Sham**			***d* (95% CI)**
**Assessment**	*N*	Mean	SD	*N*	Mean	SD		*N*	Mean	SD	*N*	Mean	SD	
**ED-related outcomes**														
BMI	16	0.11	0.73	16	-0.08	0.32	*0.33 (-0.37-1.03)*	16	0.28	1.25	16	0.04	1.05	*0.2 (-0.49-0.90)*
EDE-Q Global	16	-0.28	0.73	16	-0.4	0.79	*0.16 (-0.54-0.85)*	16	-0.43	0.83	16	-0.52	0.87	*0.1 (-0.60-0.79)*
Self-Starvation Scale	16	-4	16.15	16	-6.81	13.53	*0.19 (-0.51-0.88)*	16	-13.06	20.78	16	-9.6	13.68	*-0.2 (-0.89-0.50)*
FoFM Anxiety About Eating	16	-2.94	6.35	16	-4.44	8.97	*0.19 (-0.50-0.89)*	16	-4.69	6.39	16	-4.56	8.74	*-0.02 (-0.71-0.68)*
FoFM Food Avoidance Behaviours	16	-3.56	5.77	16	-3	3.39	*-0.12 (-0.81-0.58)*	16	-3.5	6	16	-1.69	5.51	*-0.32 (-1.01-0.39)*
FoFM Feared Concerns	16	-2	6.5	16	-1.81	9.74	*-0.02 (-0.72-0.67)*	16	-2.63	6.79	16	-1.5	9.25	*-0.14 (-0.83-0.56)*
EDRSQ Normative Eating Self-Efficacy	16	0	0.56	16	0.13	0.62	*-0.22 (-0.92-0.47)*	16	0.26	0.77	16	0.29	0.59	*-0.04 (-0.73-0.66)*
EDRSQ Body Image Self-Efficacy	16	-0.08	0.49	16	-0.11	0.61	*0.06 (-0.63-0.75)*	16	0.08	0.47	16	0.16	0.61	*-0.14 (-0.83-0.56)*
**Clinical impairments/quality of life**														
CIA	16	-6.31	12.37	16	-4.69	5.87	*-0.17 (-0.86-0.53)*	16	-9.56	15.66	16	-6	9.19	*-0.28 (-0.97-0.42)*

For peer review only

For peer review only - http://bmjopen.bmj.com/site/about/guidelines.xhtml

EQ-5D-5L: How good or bad is your health today?	8	-0.25	19.65	10	7.7	16.67	*-0.44 (-1.38-0.51)*	16	13.06	18.31	16	4.81	13.15	*0.52 (-0.19-1.22)*
Mood/affect/anxiety														
DASS-21 Depression	16	-5.13	8.94	16	-3.25	10.55	*-0.19 (-0.89-0.51)*	16	-9.13	10.61	16	-1.13	8.58	***-0.83 (-1.55- -0.10)***
DASS-21 Anxiety	16	-7.25	6.15	16	-4.13	5.44	*-0.54 (-1.24-0.17)*	16	-4.88	7.19	16	-1	4.79	*-0.63 (-1.34-0.08)*
DASS-21 Stress	16	-6.75	9.26	16	-4.5	4.82	*-0.31 (-1.00-0.40)*	16	-7.25	9.71	16	-1.63	3.88	***-0.76 (-1.47- -0.04)***
DASS-21 Total	16	-19.13	21.8	16	-11.88	17.73	*-0.37 (-1.06-0.34)*	16	-21.25	24.33	16	-3.75	12.75	***-0.9 (-1.62- -0.17)***
PANAS Positive Affect	16	1.75	5.23	16	1.06	5.4	*0.13 (-0.57-0.82)*	16	4.56	5.79	16	0.13	3.88	*0.9 (0.17-1.62)*
PANAS Negative Affect	16	-3.81	9.4	16	-1.44	5.63	*-0.31 (-1.00-0.39)*	16	-7	9.13	16	-1.94	7.42	*-0.61 (-1.31-0.11)*
POMS Total Mood Disturbance	16	-9.88	37.68	16	-8.06	21.2	*-0.06 (-0.75-0.63)*	16	-36.75	39.08	16	-5.5	20.82	***-1 (-1.73- -0.25)***
OCI-R Total	16	-3.69	7.55	16	0.94	5.58	*-0.7 (-1.41-0.02)*	16	-1.88	8.13	16	0.81	7.84	*-0.34 (-1.03-0.36)*

Bold font signifies that the confidence intervals do not include 0.

N = number; SD = standard deviation; *d* = Cohen's *d*; 95% CI = 95% confidence intervals; BMI = body mass index; EDE-Q = Eating Disorder Examination Questionnaire; FoFM = Fear of Food Measure; EDRSQ = Eating Disorder Recovery Self-Efficacy Questionnaire; EQ-5D-5L = EuroQol Quality of Life Scale; CIA = Clinical Impairment Assessment; DASS-21 = Depression, Anxiety and Stress Scale - 21 item; PANAS = Positive and Negative Affect Schedule; POMS = Profile of Mood States; OCI-R = Revised Obsessive-Compulsive Inventory

For peer review only

Additional service utilisation

At baseline, the majority of participants received outpatient treatment (n=25) and five were not receiving treatment. One participant per group received day-care treatment. A high proportion of participants were taking antidepressants and remained on this at a stable dose throughout the trial. Participant's utilisation of TAU is shown in Table 1.

At follow-up, the two participants originally in day-care treatment were instead receiving outpatient treatment. Three participants had increased treatment intensity at follow-up, with two (one per group) starting inpatient treatment and one (from the real group) starting day-care treatment. Of the remaining participants, two initially receiving no treatment started outpatient treatment and eight decreased intensity from outpatient treatment to no treatment.

Of those who completed the sham intervention, 71% took up the offer of having real rTMS treatment.

Safety

In addition to the one withdrawn participant whose weight dropped below range prior to starting treatment, one other participant's weight (from the real group) was recorded below BMI 14kg/m^2 (13.80 kg/m^2) in their final rTMS session. No other participants' weight fell below the required BMI range for the duration of treatment. Blood pressure and pulse measurements did not raise any undue concerns during the study. One participant had lowered baseline potassium and start of treatment was delayed by one week. Blood samples for the remaining participants raised no major concerns, i.e., termination or postponing of treatment was not required. For side effects experienced, see Supplementary Table 1.

DISCUSSION

Principal findings

The main findings relate to the primary feasibility objectives of this study. We were able to recruit participants as planned, after making an adjustment to recruitment numbers. Large numbers of people interested in the trial could not be recruited as travelling to South London for rTMS sessions proved impractical. A future trial therefore needs to consider offering treatment in several centres with easy transport access. Research follow-up rates exceeded our pre-specified criterion of ≥ 80%. Treatment session attendance was excellent in both groups. Although for pragmatic reasons, and compared to others, our definition of 'severe and enduring illness' was lenient [34], we managed to recruit and retain a very chronic and treatment-refractory population.

In relation to our secondary feasibility objectives, there were large between-group effect sizes on change scores from pre-treatment to follow-up on several mood variables (e.g., DASS global score d=-0.9, -1.62 to -0.17), favouring real rTMS. Comorbid depression is common in AN and has been shown to be associated with poor quality of life in people with SE-AN [4]. The importance of improving quality of life in SE-AN, rather than focussing on actively changing ED symptoms and weight gain has been emphasised [35], and the improvements in depression observed here may contribute to the broader aim of enhancing quality of life in this group. Also, given that antidepressants are typically not very effective in underweight populations or have unacceptable side effects [1], rTMS may provide an alternative treatment for common comorbid symptoms such as depression and anxiety. Within the current study, a higher proportion of participants were taking antidepressant medication (61.7%; 21/34 participants) and somewhat higher depression scores were observed, compared to other treatment studies of AN [36, 37]. This may suggest that either our participants had particularly high levels of comorbid depression or that we attracted participants who were particularly drawn to 'physical/biologically-targeted treatments' rather than psychological treatments. Having said that, many participants had previously undertaken several unsuccessful psychological treatments.

For peer review only - http://bmjopen.bmj.com/site/about/guidelines.xhtml

We considered that rTMS may be interacting with the actions of the medication to produce this antidepressant effect, however, there is no evidence for this mechanism in the depression literature. Developing better evidence for the treatment of comorbidities in EDs is a research recommendation in the recent NICE guidelines [18] and, therefore, our study potentially fills an important gap.

In addition to the mood effects, there were medium between-group effect sizes on follow-up change scores in quality of life (d=0.52, 95% CI -0.19-1.22), whereas between-group effect sizes on change scores for BMI (d=0.2, 95% CI -0.49-0.90) and ED symptoms (d=0.1, 95% CI -0.60-0.79) were small. Larger between-group effect sizes were seen on change scores from pre-treatment to follow-up than to post-treatment, suggesting that changes develop over time, rather than being due to immediate effects of rTMS. A similar delay in effect was observed in our previous case series of rTMS in SE-AN [11].

rTMS was safe, well tolerated and considered to be an acceptable treatment by participants. These various findings suggest that it is feasible to conduct a future larger-scale therapeutic RCT with a sham-controlled design to establish the therapeutic efficacy of rTMS in SE-AN.

Strengths and limitations

Our study has several strengths. It is the first RCT of multi-session rTMS treatment in individuals with AN. Secondly, it focused on people with severe, enduring illness. As such, it adds to the limited number of studies that have specifically targeted people with SE-AN. Thirdly, it was sham-controlled, which is considered the gold-standard method of evaluating the clinical efficacy of rTMS treatment in disorders such as depression [38]. Fourthly, the majority of participants did not correctly guess their treatment allocation at follow-up, suggesting blinding was successful (see Supplementary Figure 1 and 2). Lastly, the rTMS was highly individualised through the use of neuronavigation and a wide range of measures to assess relevant clinical outcomes were used.

In terms of limitations, the duration of the follow-up period was relatively short [11]. Our choices regarding the rTMS protocol and target brain area (left DLPFC) were theoretically-, evidentially- and practically-based [12]; however, the optimal brain areas to target and the rTMS protocols to administer in SE-AN are unknown. We used a shorter illness duration (minimum of 3 years) than what is commonly used to define SE-AN (e.g. 7 years [34]), but nonetheless managed to recruit participants with a long-standing illness who had typically received several previous courses of intensive treatment. Our attempts to keep researchers blind to treatment allocation were only partly successful; approximately 20% of rTMS sessions were delivered by an unblinded researcher, and another researcher correctly guessed treatment allocation of participants.

Strengths and limitations in relation to other studies

Research into treatments for people with SE-AN is limited [35]. In addition to this study, there have only been two trials with a focus on SE-AN. The first of these assessed the efficacy of 30 sessions of modified cognitive behavioural therapy for AN compared to a modified version of specialist supportive clinical management in 63 patients [6]. Between-group differences in clinical outcomes were minimal. Within-group assessments showed small to moderate effect sizes for BMI-change and medium to large for ED symptoms, depression and quality of life from baseline to end of treatment and to 6- and 12-month follow-up. The second study investigated the effects of four weeks of a synthetic cannabinoid agonist (dronabinol) versus placebo as an adjunct to a multi-modal treatment combining psychotherapy with nutritional interventions in 25 patients with SE-AN [7]. Dronabinol produced significantly greater short-term weight gain than placebo, but changes in ED symptoms were minimal during the study period. No follow-up data were reported. In both of these studies, treatment drop-out rates were low, as in the current study, highlighting the desire of people with SE-AN to participate in novel treatments.

For peer review only - http://bmjopen.bmj.com/site/about/guidelines.xhtml

Implications for future research

Building on the present study, a large-scale multi-centre RCT of real versus sham rTMS as an adjunct to TAU with a similar design, should be considered. Such a trial should include a longer follow-up period (e.g., 6- and 12-months) to assess the persistence or otherwise of rTMS effects. Secondly, a health economic analysis should be included to assess cost-effectiveness of establishing rTMS as a treatment option for SE-AN. Inclusion of inpatients with SE-AN in a future trial would be desirable, as it would be easier for them to attend daily sessions. This might also allow inclusion of patients with a BMI <14 kg/m^2, given that inpatients have regular medical monitoring and that their food intake is more regular than that of community-dwelling patients.

Several questions need to be considered in future research of rTMS in SE-AN. The optimal brain areas to target and the rTMS protocols for SE-AN are not known. High-frequency rTMS targeting the DLPFC was chosen for the current study as it was hypothesised that this would remediate the hypoactivity observed in AN in response to symptom provocation, cognitive flexibility and set-shifting tasks, and thus re-balance cognitive control and reward systems [12]. It was also selected given the strong evidence base for high-frequency DLPFC rTMS in other neurocircuit-based disorders (e.g., treatment-resistant depression [39]). Following on from research in depression, the use of low-frequency or priming low-frequency rTMS in comparison to high-frequency rTMS might be tested, as the former is thought to have fewer side effects and be more well-tolerated, with similar levels of effectiveness in the depression literature [40, 41]. Future studies should also consider rTMS as an adjunct to psychological therapies [42]. Other neuromodulation treatments in combination with cognitive interventions have shown promise [43, 44], and so, addition of rTMS to structured psychotherapy or cognitive/behavioural tasks in SE-AN may help increase its efficacy [12]. Finally, additional work on neural and neurocognitive mechanisms of action of rTMS and the cost-effectiveness of this treatment are necessary.

CONCLUSION

In this feasibility RCT, rTMS was safe and well tolerated. This study provides preliminary evidence for the therapeutic potential of rTMS treatment in community-dwelling SE-AN as an adjunct to TAU. It suggests that it is feasible to conduct a future larger-scale therapeutic RCT with a sham-controlled design to establish/confirm the therapeutic efficacy of rTMS in AN. The findings from this trial will inform a future large-scale RCT with respect to decisions on primary outcome measures and other aspects of protocol development, such as sample size, design, location and number of research centres. Future studies should include a longer follow-up period and a formal assessment of cost-effectiveness. Consideration should also be given to use of alternative stimulation protocols (e.g., low-frequency rTMS) and the combination of rTMS and ED-specific therapies/tasks to maximise impact upon ED and mood.

For peer review only - http://bmjopen.bmj.com/site/about/guidelines.xhtml

ACKNOWLEDGEMENTS
We would like to thank the individuals who participated in this study for their time and commitment. The authors would like to thank Daniela Mercado Beivide and Pia Lauffer for their assistance in data collection, the radiographers at the KCL Centre for Neuroimaging Sciences for their work during with the MRI scans and their guidance on decisions regarding eligibility and protocol development, and the phlebotomy staff at South London and Maudsley Outpatients'. This study was supported by the United Kingdom Clinical Research Collaboration-registered King's Clinical Trials Unit at King's Health Partners, which is part funded by the NIHR Biomedical Research Centre for Mental Health at South London and Maudsley NHS Foundation Trust and King's College London and the NIHR Evaluation, Trials and Studies Coordinating Centre.

CONTRIBUTORS

BD, SB, JM and US drafted the manuscript. BD and SB conducted data analysis, which was supervised by BC and US. OOD, ICC, MK, SJR, NK, DG and ASD revised the manuscript critically for important intellectual content. Ethical approval was obtained by SB, JM, MK and US. Funding from NIHR was obtained by US, JM and ICC. Funding from the NIHR BRC was obtained by SB, JM, MK, OOD, ICC and US. JM, SB and US registered the trial on the ISRCTN registry. JM, SB, MK, US and BD were involved in participant recruitment. BD, SB, JM, MK, JW and SJR were involved in data collection. rTMS treatment was provided by BD, SB, JM and MK. OOD, MK, JW, SJR, SB, JM, ICC and US contributed to the design and conception of the study. All authors, were involved in drafting, critiquing and approving of the manuscript, and accept responsibility for the accuracy and integrity of this work.

Registration
The trial is registered on the ISRCTN registry. Registration number: ISRCTN14329415 (DOI: 10.1186/ISRCTN14329415). Date of registration: 23 July 2015

Protocol
The study protocol was published prior to recruitment [16].

Funding
This work was supported by a National Institute for Health Research (NIHR) Research for Patient Benefit (RfPB) grant (RB-PG-1013-32049) and Infrastructure Support for Pilot studies from the NIHR Biomedical Research Centre (BRC) at South London and Maudsley NHS Foundation Trust (SLaM) and King's College London (KCL). This paper presents independent research funded by the National Institute for Health Research (NIHR) under its Research for Patient Benefit (RfPB) Programme (Grant Reference Number PB-PG-1013-32049). The views expressed are those of the author(s) and not necessarily those of the NHS, the NIHR or the Department of Health.

Competing Interests
None declared.

Data sharing statement
No additional data are available.

For peer review only - http://bmjopen.bmj.com/site/about/guidelines.xhtml

REFERENCES

1. Zipfel S, Giel KE, Bulik CM, et al. Anorexia nervosa: aetiology, assessment, and treatment. *Lancet Psychiatry* 2015;2:1099-111.
2. Treasure J, Zipfel S, Micali N, et al. Anorexia nervosa. *Nat Rev Dis Primers* 2015;1:15074.
3. Steinhausen HC. The outcome of anorexia nervosa in the 20th century. *Am J Psychiatry* 2002;159:1284-93.
4. Arkell J, Robinson P. A pilot case series using qualitative and quantitative methods: biological, psychological and social outcome in severe and enduring eating disorder (anorexia nervosa). *Int J Eat Disord* 2008;41:650-6.
5. Brockmeyer T, Friederich HC, Schmidt U. Advances in the treatment of anorexia nervosa: a review of established and emerging interventions. *Psychol Med* 2017:1-37.
6. Touyz S, Le Grange D, Lacey H, et al. Treating severe and enduring anorexia nervosa: a randomized controlled trial. *Psychol Med* 2013;43:2501-11.
7. Andries A, Frystyk J, Flyvbjerg A, et al. Dronabinol in severe, enduring anorexia nervosa: a randomized controlled trial. *Int J Eat Disord* 2014;47:18-23.
8. Hay PJ, Touyz S, Sud R. Treatment for severe and enduring anorexia nervosa: a review. *Aust N Z J Psychiatry* 2012;46:1136-44.
9. Dalton B, Campbell IC, Schmidt U. Neuromodulation and neurofeedback treatments in eating disorders and obesity. *Curr Opin Psychiatry* 2017;30:458-73.
10. Choudhary P, Roy P, Kumar Kar S. Improvement of weight and attitude towards eating behaviour with high frequency rTMS augmentation in anorexia nervosa. *Asian J Psychiatr* 2017;28:160.
11. McClelland J, Kekic M, Campbell IC, et al. Repetitive transcranial magnetic stimulation (rTMS) treatment in enduring anorexia nervosa: a case series. *Eur Eat Disord Rev* 2016;24:157-63.
12. Dunlop KA, Woodside B, Downar J. Targeting neural endophenotypes of eating disorders with non-invasive brain stimulation. *Front Neurosci* 2016;10:30.
13. McClelland J, Kekic M, Bozhilova N, et al. A randomised controlled trial of neuronavigated repetitive transcranial magnetic stimulation (rTMS) in anorexia nervosa. *PLOS One* 2016;11:e0148606.
14. Van den Eynde F, Guillaume S, Broadbent H, et al. Repetitive transcranial magnetic stimulation in anorexia nervosa: a pilot study. *Eur Psychiatry* 2013;28:98-101.
15. McClelland J, Bozhilova N, Nestler S, et al. Improvements in symptoms following neuronavigated repetitive transcranial magnetic stimulation (rTMS) in severe and enduring anorexia nervosa: findings from two case studies. *Eur Eat Disord Rev* 2013;21:500-6.
16. Bartholdy S, McClelland J, Kekic M, et al. Clinical outcomes and neural correlates of 20 sessions of repetitive transcranial magnetic stimulation in severe and enduring anorexia nervosa (the TIARA study): study protocol for a randomised controlled feasibility trial. *Trials* 2015;16:548.
17. American Psychiatric Association. Diagnostic and Statistical Manual of Mental Disorders (5th ed.). Arlington, VA: American Psychiatric Publishing, 2013.
18. National Institute for Health and Care Excellence. Eating disorders: recognition and treatment. NICE Clinical Guideline NG69. 2017. https://www.nice.org.uk/guidance/ng69 (accessed 27th November 2017).
19. Fitzgerald PB, Hoy K, McQueen S, et al. A randomized trial of rTMS targeted with MRI based neuro-navigation in treatment-resistant depression. *Neuropsychopharmacology* 2009;34:1255-62.
20. Fairburn C. Appendix: Eating Disorder Examination Questionnaire (EDE-Q Version 6.0) Cognitive Behaviour Therapy and Eating Disorders. New York: Guilford Press, 2008.
21. Levinson CA, Byrne M. The fear of food measure: a novel measure for use in exposure therapy for eating disorders. *Int J Eat Disord* 2015;48:271-83.
22. Godier LR, Park RJ. A novel measure of compulsive food restriction in anorexia nervosa: validation of the Self-Starvation Scale (SS). *Eat Behav* 2015;17:10-13.
23. Marinilli Pinto A, Guarda AS, Heinberg LJ, et al. Development of the eating disorder recovery self-efficacy questionnaire. *Int J Eat Disord* 2006;39:376-84.

For peer review only - http://bmjopen.bmj.com/site/about/guidelines.xhtml

24. Lovibond S, Lovibond P. Manual for the Depression Anxiety Stress Scales. 2nd ed. Sydney: Psychology Foundation, 1995.
25. Watson D, Clark LA, Tellegen A. Development and validation of brief measures of positive and negative affect: the PANAS scales. *J Pers Soc Psychol* 1988;54:1063-70.
26. McNair D, Lorr M, Droppleman L. Profile of Mood States Manual. San Diego: Educational and Industrial Testing Services, 1992.
27. Foa EB, Huppert JD, Leiberg S, et al. The Obsessive-Compulsive Inventory: development and validation of a short version. *Psychol Assess* 2002;14:485-96.
28. Herdman M, Gudex C, Lloyd A, et al. Development and preliminary testing of the new five-level version of EQ-5D (EQ-5D-5L). *Qual Life Res* 2011;20:1727-36.
29. Bohn K, Doll HA, Cooper Z, et al. The measurement of impairment due to eating disorder psychopathology. *Behav Res Ther* 2008;46:1105-10.
30. Bohn K, Fairburn CG. The Clinical Impairment Assessment questionnaire (CIA). Cognitive behavioral therapy for eating disorders. New York: Guilford Press, 2008.
31. Beecham J, Knapp M. Costing psychiatric interventions. In: Thornicroft G, ed. Measuring mental health needs. London: Gaskell, 2001:200-24.
32. Rossi S, Hallett M, Rossini PM, et al. Safety, ethical considerations, and application guidelines for the use of transcranial magnetic stimulation in clinical practice and research. *Clin Neurophysiol* 2009;120:2008-39.
33. Lancaster GA, Dodd S, Williamson PR. Design and analysis of pilot studies: recommendations for good practice. *J Eval Clin Pract* 2004;10:307-12.
34. Broomfield C, Stedal K, Touyz S, et al. Labeling and defining severe and enduring anorexia nervosa: a systematic review and critical analysis. *Int J Eat Disord* 2017;50:611-23.
35. Wonderlich S, Mitchell JE, Crosby RD, et al. Minimizing and treating chronicity in the eating disorders: a clinical overview. *Int J Eat Disord* 2012;45:467-75.
36. Schmidt U, Magill N, Renwick B, et al. The Maudsley Outpatient Study of Treatments for Anorexia Nervosa and Related Conditions (MOSAIC): Comparison of the Maudsley Model of Anorexia Nervosa Treatment for Adults (MANTRA) with specialist supportive clinical management (SSCM) in outpatients with broadly defined anorexia nervosa: A randomized controlled trial. *J Consult Clinl Psychol* 2015;83:796.
37. Byrne S, Wade T, Hay P, et al. A randomised controlled trial of three psychological treatments for anorexia nervosa. *Psychol Med* 2017:1-11.
38. Gaynes BN, Lloyd SW, Lux L, et al. Repetitive transcranial magnetic stimulation for treatment-resistant depression: a systematic review and meta-analysis. *J Clin Psychiatry* 2014;75:477-89; quiz 89.
39. Leggett LE, Soril LJ, Coward S, et al. Repetitive Transcranial Magnetic Stimulation for Treatment-Resistant Depression in Adult and Youth Populations: A Systematic Literature Review and Meta-Analysis. *Prim Care Companion CNS Disord* 2015;17.
40. Brunelin J, Jalenques I, Trojak B, et al. The efficacy and safety of low frequency repetitive transcranial magnetic stimulation for treatment-resistant depression: the results from a large multicenter French RCT. *Brain Stimul* 2014;7:855-63.
41. Fitzgerald PB, Brown TL, Marston NA, et al. Transcranial magnetic stimulation in the treatment of depression: a double-blind, placebo-controlled trial. *Arch Gen Psychiatry* 2003;60:1002-8.
42. Bajbouj M, Padberg F. A perfect match: noninvasive brain stimulation and psychotherapy. *Eur Arch Psychiatry Clin Neurosci* 2014;264 Suppl 1:S27-33.
43. De Raedt R, Vanderhasselt MA, Baeken C. Neurostimulation as an intervention for treatment resistant depression: From research on mechanisms towards targeted neurocognitive strategies. *Clin Psychol Rev* 2015;41:61-9.
44. Elmasry J, Loo C, Martin D. A systematic review of transcranial electrical stimulation combined with cognitive training. *Restor Neurol Neurosci* 2015;33:263-78.

For peer review only

For peer review only - http://bmjopen.bmj.com/site/about/guidelines.xhtml

FIGURES

Figure 1. Consort diagram of participant involvement.

Supplementary File, Figure 1. Blinding guesses combined with level of certainty for all participants in the real rTMS intervention group.

Supplementary File, Figure 2. Blinding guesses combined with level of certainty for all participants in the sham rTMS intervention group.

For peer review only

For peer review only - http://bmjopen.bmj.com/site/about/guidelines.xhtml

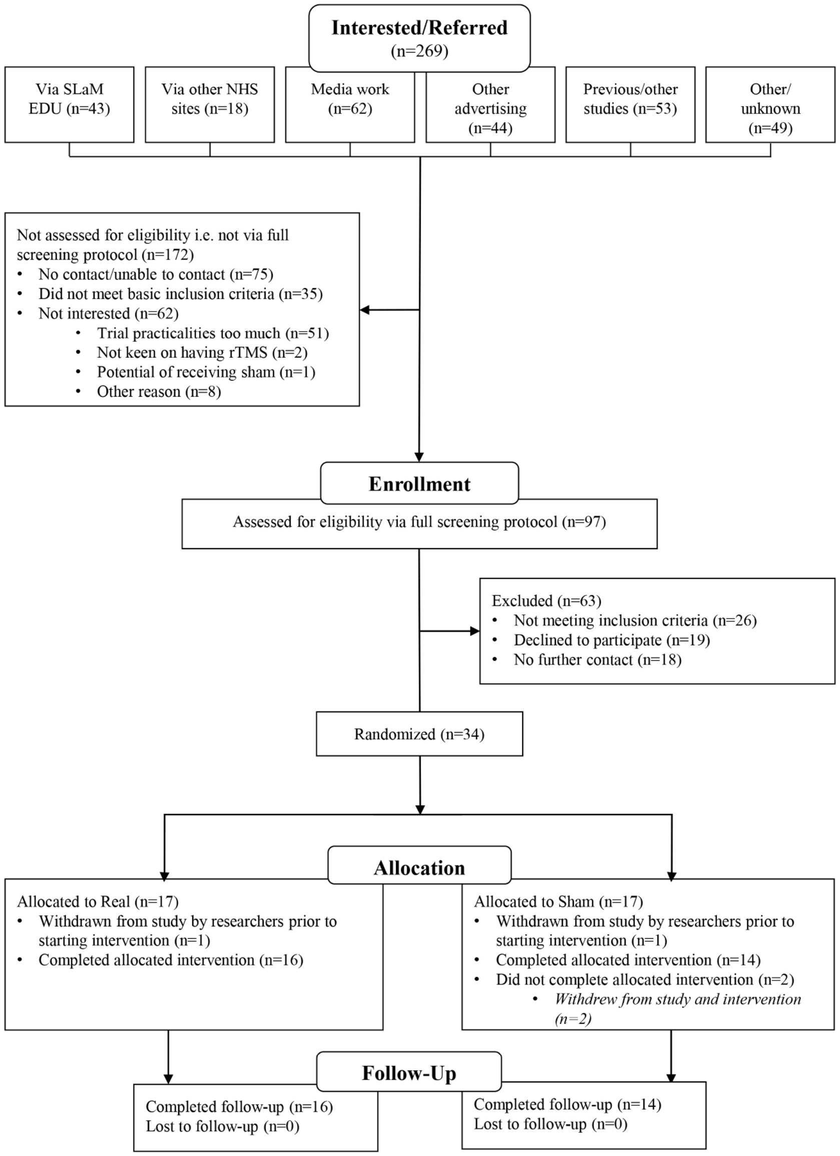

Figure 1. Consort diagram of participant involvement.

234x330mm (300 x 300 DPI)

A randomised controlled feasibility trial of real versus sham repetitive transcranial magnetic stimulation treatment in adults with severe and enduring anorexia nervosa: the TIARA study.

Dalton, Bartholdy, McClelland et al.

SUPPLEMENTARY METHODS

Blinding

Allocation concealment:

Unblinded treatment arm allocations were sent to a member of research staff who was independent from the trial team and had no contact with participants. Prior to trial commencement, this researcher labelled the real and sham rTMS coils with a coloured (red/green) keyring to blind the rTMS therapists to treatment allocation. This researcher then converted the unblinded randomisation allocation into the blinded treatment codes (red/green), and passed on this blinded allocation information to the rTMS therapists.

Assessment of blinding success:

Following a participant's rTMS treatment (at approximately 1 month post-randomisation), all researchers involved in their treatment provided a guess as to the participant's treatment allocation, and indicated certainty using a 10cm visual analogue (VAS) scale.

Every 5 sessions, participants were asked to guess their treatment allocation and record their certainty in their guess using a 10cm VAS scale.

Safety, tolerability and participants' experience of treatment

Assessment of side-effects:

rTMS-associated side effects were assessed at the beginning of each session (except session 1), with participants reporting on a 10cm visual analogue scale (VAS) whether they had experienced any of the following common rTMS side effects: headache, discomfort on the head, nausea, dizziness, light headedness, skin irritation or drowsiness. Participants were also able to record any other side effects they experienced.

Participants' expectations and experience of treatment:

Semi-structured qualitative interviews assessing treatment expectations and experience were conducted at follow-up (4-months post-randomisation). Findings will be reported in full elsewhere. A VAS of participants' expectations regarding the effects of rTMS on their condition (0 = 'I expect to feel much worse' and 10 = 'I expect to feel much better') was carried out in their first rTMS session.

SUPPLEMENTARY RESULTS

Blinding

Participant blinding:

Profile plots of participants' blinding guess combined with their rating of certainty are presented below separately for the real (see Figure 1) and sham (see Figure 2) intervention group. Each line in the profile plot refers to a different participant, the think black line provides the median. A data point in the top part of the graph reflects that the participant guessed real. A data point in the bottom part of the graph reflects that the participant guessed sham. Certainty scores are reflected on the scale of 0 - 10/-10, with 0 meaning that the participant was completely unsure of their blinding decision and 10/10 meaning the participant was completely sure of their blinding decision.

For peer review only - http://bmjopen.bmj.com/site/about/guidelines.xhtml

A randomised controlled feasibility trial of real versus sham repetitive transcranial magnetic stimulation treatment in adults with severe and enduring anorexia nervosa: the TIARA study.

Dalton, Bartholdy, McClelland et al.

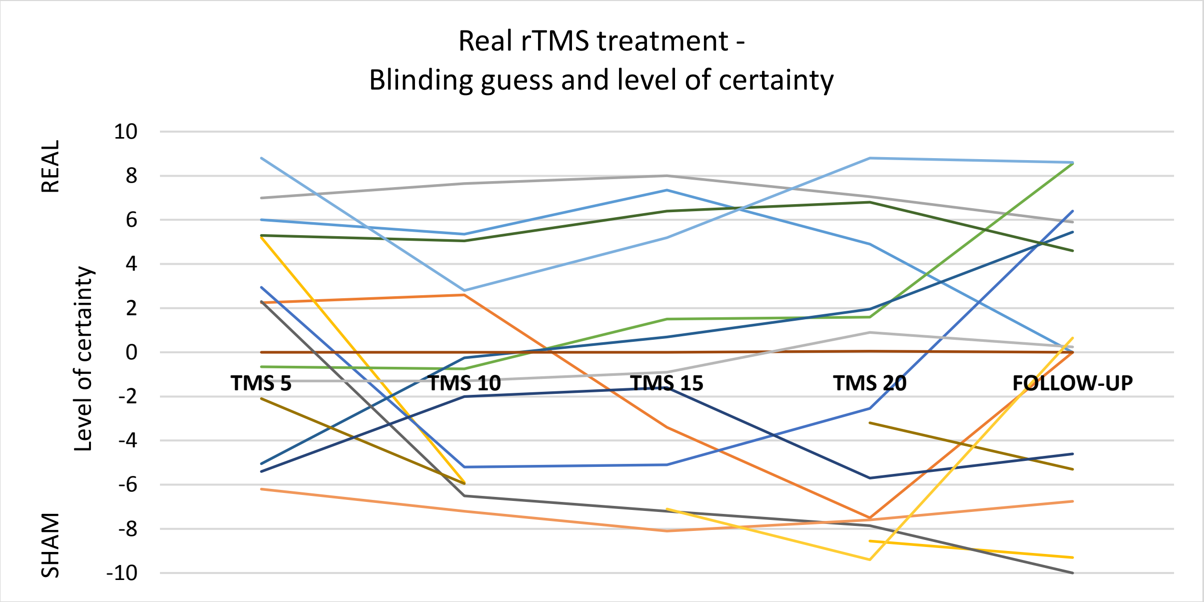

Figure 1. Blinding guesses combined with level of certainty for all participants in the real rTMS intervention group.

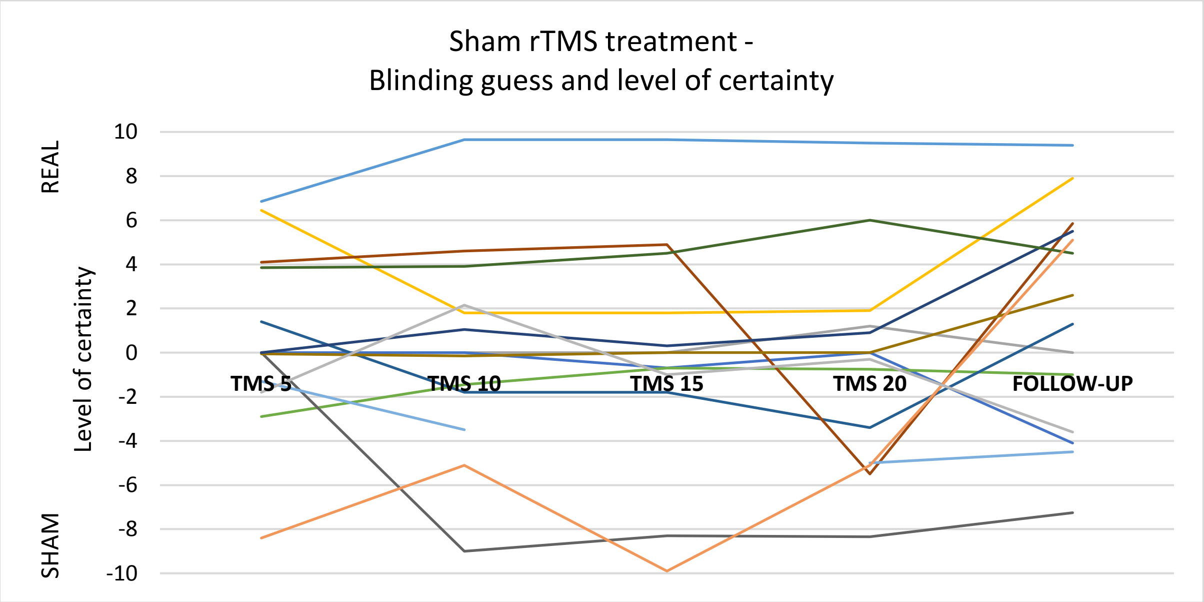

Figure 2. Blinding guesses combined with level of certainty for all participants in the sham rTMS intervention group.

Researcher blinding:

The rTMS therapists provided an allocation guess for each participant that they had administered rTMS to, once the participant had completed treatment. JM provided blinding guesses on n=17, correctly guessing the allocation of 41% of these participants. SB provided blinding guesses on n=29, correctly guessing the allocation of 31% of these participants. MK providing blinding guesses on

n=26, correctly guessing the allocation of 100% of these participants. Generally, certainty in their blinding guesses increased as the trial progressed.

Safety, tolerability, side effects and participants' expectations and experience of treatment

Tolerability and side effects:
Mean frequency and maximum intensity/severity of reported side effects are presented in Supplementary Table 1. In both groups, headaches were the most frequently reported side effect, followed by drowsiness in the real group and nausea in the sham group. Generally, the intensity/severity of side effects reduced as rTMS sessions progressed. Mean frequency of side effects was slightly higher in the real group compared to the sham group; however, the sham group experienced the same range of side effects and some had greater reported intensity/severity (e.g., headaches, nausea, dizziness, light-headedness). One participant reported chest pains as a side effect. Sessions were postponed and resumed after two days once the participant was medically cleared.

Treatment expectations:
The mean rating of participants' expectation of the efficacy of rTMS sessions in improving their condition was 6.63 (SD 1.78, range 1.8-10), with the majority of participants (n=27) expecting some clinical improvement (rating of 5-10).

Treatment experience:
Preliminary analysis of the qualitative interviews (full analyses of these data will be presented elsewhere; NB 29 transcripts available, *n*=1 transcript missing due to technical problems with recording) of patient treatment experiences suggests that many participants found attending the rTMS clinic a "very positive" and "really interesting" experience and they "enjoyed the sessions" (22/29 participants). Several participants (14/29) highlighted practical difficulties and the commitment involved: "it was quite hard travelling up every day", "it would be easier if you didn't have to do it every day", "took a lot of time and commitment"; however, some found it "much easier" than they thought it would be once they "got used to the routine" (7/29). Participants (18/29) also reported that whilst rTMS initially felt uncomfortable due to the tapping sensation on the head, any discomfort experienced lessened over time as they "got used to it".

For peer review only

A randomised controlled feasibility trial of real versus sham repetitive transcranial magnetic stimulation treatment in adults with severe and enduring anorexia nervosa: the TIARA study.

Dalton, Bartholdy, McClelland et al.

	Real					Sham				
**Side effects**	*Total*	Sessions 2-5	Sessions 6-10	Sessions 11-15	Sessions 16-20	*Total*	Sessions 2-5	Sessions 6-10	Sessions 11-15	Sessions 16-20
Frequency of experience side effects(Mean (SD))										
Headache	13.25 (16.08)	2.75 (1.44)	3.88 (1.41)	3.50 (1.97)	3.13 (1.78)	8.25 (6.96)	2.56 (1.50)	2.50 (2.25)	1.69 (2.02)	1.50 (2.00)
Discomfort on head	9.19 (8.07)	2.00 (1.55)	2.50 (2.31)	2.56 (2.39)	2.13 (2.22)	5.44 (7.24)	2.00 (1.71)	1.44 (2.22)	1.06 (1.98)	0.94 (2.02)
Nausea	8.19 (7.48)	1.75 (1.65)	2.31 (2.33)	2.31 (2.12)	1.81 (1.94)	7.31 (7.65)	2.19 (7.65)	2.06 (1.38)	1.63 (2.36)	1.44 (2.25)
Dizziness	7.44 (7.62)	1.63 (1.75)	2.13 (2.31)	1.88 (2.06)	1.81 (2.07)	5.50 (7.13)	1.75 (1.73)	1.56 (2.10)	1.25 (1.95)	0.94 (2.02)
Light-headedness	8.13 (7.54)	1.81 (1.72)	2.13 (2.19)	2.31 (2.18)	1.88 (2.06)	6.69 (7.42)	2.00 (1.71)	1.88 (2.25)	1.50 (2.00)	1.31 (2.02)
Skin irritation	5.75 (6.82)	1.25 (1.39)	1.31 (1.74)	1.63 (2.16)	1.56 (1.97)	4.63 (7.40)	1.25 (1.73)	1.50 (2.31)	0.96 (1.88)	0.94 (2.02)
Drowsiness	10.38 (7.56)	2.25 (1.57)	3.00 (2.16)	2.88 (2.22)	2.25 (2.18)	7.06 (7.09)	1.88 (1.71)	2.38 (2.13)	1.38 (2.09)	1.44 (2.03)
Maximum intensity / severity of experienced side effects (Mean (SD))										
Headache	4.93 (2.29)	3.01 (2.69)	3.86 (2.33)	2.04 (2.06)	1.99 (2.42)	5.16 (2.77)	4.7 (3.05)	3.22 (3.00)	1.52 (1.83)	1.16 (1.84)
Discomfort on head	3.22 (3.26)	2.78 (3.11)	1.95 (3.07)	0.73 (0.96)	0.77 (1.62)	2.93 (3.14)	3.05 (3.21)	0.69 (1.80)	0.13 (0.29)	0.05 (0.16)
Nausea	2.91 (3.18)	2.34 (2.81)	1.67 (2.91)	0.58 (0.79)	0.91 (2.11)	3.46 (3.11)	2.42 (2.73)	1.34 (2.70)	1.46 (2.81)	1.09 (2.31)
Dizziness	1.70 (2.16)	1.08 (1.98)	0.99 (1.92)	0.56 (1.63)	0.91 (1.86)	3.29 (2.96)	2.05 (3.04)	1.05 (1.63)	1.31 (1.98)	0.19 (0.49)
Light-headedness	2.33 (2.60)	1.53 (2.15)	1.54 (2.44)	1.11 (2.09)	0.73 (1.73)	2.95 (3.01)	2.13 (2.97)	1.29 (2.18)	1.76 (2.69)	0.42 (0.75)
Skin irritation	0.81 (1.15)	0.39 (0.59)	0.45 (0.83)	0.34 (0.89)	0.33 (0.89)	0.68 (2.05)	0.44 (1.25)	0.67 (2.13)	0.19 (0.57)	0.05 (0.13)
Drowsiness	5.23 (3.60)	4.06 (3.41)	3.94 (3.62)	2.38 (2.80)	2.05 (2.58)	3.51 (3.16)	2.94 (3.28)	2.89 (2.74)	1.27 (2.59)	1.26 (2.14)

Supplementary Table 1. Mean frequency and mean maximum intensity / severity of experienced side effects.

SD = standard deviation

For peer review only

For peer review only - http://bmjopen.bmj.com/site/about/guidelines.xhtml

CONSORT 2010 checklist of information to include when reporting a pilot or feasibility trial*

Section/Topic	Item No	Checklist item	Reported on page No
**Title and abstract**			
	1a	Identification as a pilot or feasibility randomised trial in the title	1
	1b	Structured summary of pilot trial design, methods, results, and conclusions (for specific guidance see CONSORT abstract extension for pilot trials)	2
**Introduction**			
Background and objectives	2a	Scientific background and explanation of rationale for future definitive trial, and reasons for randomised pilot trial	3
	2b	Specific objectives or research questions for pilot trial	3
**Methods**			
Trial design	3a	Description of pilot trial design (such as parallel, factorial) including allocation ratio	4
	3b	Important changes to methods after pilot trial commencement (such as eligibility criteria), with reasons	6
Participants	4a	Eligibility criteria for participants	4
	4b	Settings and locations where the data were collected	4
	4c	How participants were identified and consented	4
Interventions	5	The interventions for each group with sufficient details to allow replication, including how and when they were actually administered	4
Outcomes	6a	Completely defined prespecified assessments or measurements to address each pilot trial objective specified in 2b, including how and when they were assessed	5
	6b	Any changes to pilot trial assessments or measurements after the pilot trial commenced, with reasons	N/A
	6c	If applicable, prespecified criteria used to judge whether, or how, to proceed with future definitive trial	5
Sample size	7a	Rationale for numbers in the pilot trial	6
	7b	When applicable, explanation of any interim analyses and stopping guidelines	6
Randomisation:			
Sequence generation	8a	Method used to generate the random allocation sequence	4
	8b	Type of randomisation(s); details of any restriction (such as blocking and block size)	4
Allocation concealment mechanism	9	Mechanism used to implement the random allocation sequence (such as sequentially numbered containers), describing any steps taken to conceal the sequence until interventions were assigned	Supplementary file, page 1

For peer review only

Implementation	10	Who generated the random allocation sequence, who enrolled participants, and who assigned participants to interventions	4 and Supplementary file, page 1
Blinding	11a	If done, who was blinded after assignment to interventions (for example, participants, care providers, those assessing outcomes) and how	4
	11b	If relevant, description of the similarity of interventions	5
Statistical methods	12	Methods used to address each pilot trial objective whether qualitative or quantitative	6
**Results**			
Participant flow (a diagram is strongly recommended)	13a	For each group, the numbers of participants who were approached and/or assessed for eligibility, randomly assigned, received intended treatment, and were assessed for each objective	6 and Figure 1
	13b	For each group, losses and exclusions after randomisation, together with reasons	6 and Figure 1
Recruitment	14a	Dates defining the periods of recruitment and follow-up	6
	14b	Why the pilot trial ended or was stopped	6
Baseline data	15	A table showing baseline demographic and clinical characteristics for each group	7
Numbers analysed	16	For each objective, number of participants (denominator) included in each analysis. If relevant, these numbers should be by randomised group	7
Outcomes and estimation	17	For each objective, results including expressions of uncertainty (such as 95% confidence interval) for any estimates. If relevant, these results should be by randomised group	8/9
Ancillary analyses	18	Results of any other analyses performed that could be used to inform the future definitive trial	8
Harms	19	All important harms or unintended effects in each group (for specific guidance see CONSORT for harms)	11
	19a	If relevant, other important unintended consequences	N/A
**Discussion**			
Limitations	20	Pilot trial limitations, addressing sources of potential bias and remaining uncertainty about feasibility	12
Generalisability	21	Generalisability (applicability) of pilot trial methods and findings to future definitive trial and other studies	12
Interpretation	22	Interpretation consistent with pilot trial objectives and findings, balancing potential benefits and harms, and considering other relevant evidence	11
	22a	Implications for progression from pilot to future definitive trial, including any proposed amendments	12
**Other information**			
Registration	23	Registration number for pilot trial and name of trial registry	14
Protocol	24	Where the pilot trial protocol can be accessed, if available	14
Funding	25	Sources of funding and other support (such as supply of drugs), role of funders	14

For peer review only

	26	Ethical approval or approval by research review committee, confirmed with reference number	4

Citation: Eldridge SM, Chan CL, Campbell MJ, Bond CM, Hopewell S, Thabane L, et al. CONSORT 2010 statement: extension to randomised pilot and feasibility trials. BMJ. 2016;355.
*We strongly recommend reading this statement in conjunction with the CONSORT 2010, extension to randomised pilot and feasibility trials, Explanation and Elaboration for important clarifications on all the items. If relevant, we also recommend reading CONSORT extensions for cluster randomised trials, non-inferiority and equivalence trials, non-pharmacological treatments, herbal interventions, and pragmatic trials. Additional extensions are forthcoming: for those and for up to date references relevant to this checklist, see www.consort-statement.org.

For peer review only

BMJ Open

A randomised controlled feasibility trial of real versus sham repetitive transcranial magnetic stimulation treatment in adults with severe and enduring anorexia nervosa: the TIARA study.

Journal:	*BMJ Open*
Manuscript ID	bmjopen-2018-021531.R1
Article Type:	Research
Date Submitted by the Author:	09-Apr-2018
Complete List of Authors:	Dalton, Bethan; Institute of Psychiatry, Psychology & Neuroscience, King's College London, Section of Eating Disorders, Department of Psychological Medicine<br>Bartholdy, Savani; Institute of Psychiatry, Psychology & Neuroscience, King's College London, Section of Eating Disorders, Department of Psychological Medicine<br>McClelland, Jessica; Institute of Psychiatry, Psychology & Neuroscience, King's College London, Section of Eating Disorders, Department of Psychological Medicine<br>Kekic, Maria; Institute of Psychiatry, Psychology & Neuroscience, King's College London, Section of Eating Disorders, Department of Psychological Medicine<br>Rennalls, Samantha; Institute of Psychiatry, Psychology & Neuroscience, King's College London, Section of Eating Disorders, Department of Psychological Medicine<br>Werthmann, Jessica; Institute of Psychiatry, Psychology & Neuroscience, King's College London, Section of Eating Disorders, Department of Psychological Medicine<br>Carter, B; Institute of Psychiatry, Psychology & Neuroscience, King's College London, Department of Biostatistics and Health Informatics<br>O'Daly, Owen; Institute of Psychiatry, Psychology & Neuroscience, King's College London, Centre for Neuroimaging Sciences, Department of Neuroimaging<br>Campbell, Iain; Institute of Psychiatry, Psychology & Neuroscience, King's College London, Section of Eating Disorders, Department of Psychological Medicine<br>David, Anthony; Institute of Psychiatry, Psychology & Neuroscience, King's College London, Department of Psychosis Studies<br>Glennon, Danielle; South London and Maudsley NHS Foundation Trust<br>Kern, Nikola; South London and Maudsley NHS Foundation Trust<br>Schmidt, Ulrike; Institute of Psychiatry, Psychology & Neuroscience, King's College London, Section of Eating Disorders, Department of Psychological Medicine
**Primary Subject Heading**:	Mental health
Secondary Subject Heading:	Mental health

Keywords:	Eating disorders < PSYCHIATRY, Neuromodulation, Repetitive transcranial magnetic stimulation (rTMS), Anorexia nervosa

SCHOLARONE™ Manuscripts

For peer review only

A randomised controlled feasibility trial of real versus sham repetitive transcranial magnetic stimulation treatment in adults with severe and enduring anorexia nervosa: the TIARA study.

Bethan Dalton[1]*, Savani Bartholdy[1]*, Jessica McClelland[1]*, Maria Kekic[1], Samantha J. Rennalls[2], Jessica Werthmann[1], Ben Carter[3], Owen G. O'Daly[2], Iain C. Campbell[1], Anthony S. David[4], Danielle Glennon[5], Nikola Kern[5] and Ulrike Schmidt[1,5#]

1. Section of Eating Disorders, Department of Psychological Medicine, Institute of Psychiatry, Psychology & Neuroscience, King's College London, London, UK

2. Centre for Neuroimaging Sciences, Department of Neuroimaging, Institute of Psychiatry, Psychology & Neuroscience, King's College London, London, UK

3. Department of Biostatistics and Health Informatics, Institute of Psychiatry, Psychology & Neuroscience, King's College London, London, UK

4. Department of Psychosis Studies, Institute of Psychiatry, Psychology & Neuroscience, King's College London, London, UK.

5. South London and Maudsley NHS Foundation Trust, London, UK

*Authors contributed equally

# Corresponding author: Professor Ulrike Schmidt, PO59 Section of Eating Disorders, Department of Psychological Medicine, Institute of Psychiatry, Psychology and Neuroscience, King's College London, London, SE5 8AF, UK. ulrike.schmidt@kcl.ac.uk.

Word count: 4468

For peer review only - http://bmjopen.bmj.com/site/about/guidelines.xhtml

ABSTRACT

Objective: Treatment options for severe, enduring anorexia nervosa (SE-AN) are limited. Non-invasive neuromodulation is a promising emerging intervention. Our study is a feasibility randomised controlled trial (RCT) of repetitive transcranial magnetic stimulation (rTMS) in individuals with SE-AN, which aims to inform the design of a future large-scale trial.

Design: Double-blind, parallel group, two-arm, sham-controlled trial.

Setting: Specialist eating disorders centre.

Participants: Community-dwelling people with AN, an illness duration of ≥3 years and at least one previous completed treatment.

Interventions: Participants received 20 sessions (administered over 4 weeks) of MRI-guided real or sham high-frequency rTMS to the left dorsolateral prefrontal cortex in addition to treatment-as-usual (TAU).

Outcomes: Primary outcomes were recruitment, attendance and retention rates. Secondary outcomes included body mass index [BMI], eating disorder symptoms, mood, quality of life, and rTMS safety and tolerability. Assessments were conducted at baseline, post-treatment and follow-up (i.e., at 0-, 1- and 4-months post-randomisation).

Results: Thirty-four participants (17 per group) were randomly allocated to real or sham rTMS. One participant per group was withdrawn prior to the intervention due to safety concerns. Two participants (both receiving sham) did not complete the treatment. rTMS was safe and well tolerated. Between-group effect sizes of change scores (baseline to follow-up) were small for BMI (d=0.2, 95% CI -0.49-0.90) and eating disorder symptoms (d=0.1, 95% CI -0.60-0.79), medium for quality of life and moderate to large (d=0.61 to 1.0) for mood outcomes, all favouring rTMS over sham.

Conclusions: The treatment protocol is feasible and acceptable to participants. Outcomes provide preliminary evidence for the therapeutic potential of rTMS in SE-AN. Largest effects were observed on variables assessing mood. This study supports the need for a larger confirmatory trial to evaluate the effectiveness of multi-session rTMS in SE-AN. Future studies should include a longer follow-up period and an assessment of cost-effectiveness.

Registration: The trial is registered on the ISRCTN registry. Registration number: ISRCTN14329415 (DOI: 10.1186/ISRCTN14329415). Date of registration: 23 July 2015.

Keywords
Eating disorders; neuromodulation; anorexia nervosa; repetitive transcranial magnetic stimulation (rTMS)

Strengths and limitations of this study

- This is the first randomised controlled feasibility trial of multi-session repetitive transcranial magnetic stimulation (rTMS) treatment people with anorexia nervosa (AN).
- It targeted those with severe and enduring AN (SE-AN).
- A range of outcomes were assessed (recruitment, retention, safety, tolerability and effect sizes of clinical outcome variables) and thus it provides useful data for implementing a larger scale randomised controlled trial of rTMS in SE-AN.
- The study had a small sample size, was not powered or designed to assess the efficacy of rTMS in SE-AN and the follow-up duration was short.

For peer review only - http://bmjopen.bmj.com/site/about/guidelines.xhtml

INTRODUCTION

Anorexia nervosa (AN) is a life-threatening disorder of multi-factorial aetiology. Alterations in neural circuits involved in reward processing, negative affect and stress, appetite regulation, cognitive (self-regulatory) control and socio-emotional processes have been implicated in its causation and maintenance [1, 2].

Approximately 20% of patients develop a severe, enduring form of illness (SE-AN) [3]. These patients typically have high levels of depression and anxiety, are socially isolated and markedly impaired in their ability to care for themselves. In fact, their quality of life is comparable to that of patients with depression and impairments in social contact and self-care are comparable to those in psychosis [4]. Recent research on treatments for AN has mainly focused on psychological therapies [5]. Only two small trials have focused on SE-AN, using psychological therapies or medication to improve quality of life [6] or achieve weight gain, albeit with limited success [7]. Thus, there is a need for novel interventions for this group [6, 8, 5, 1].

Non-invasive neuromodulation is a promising emerging treatment for SE-AN [5, 9, 2], in particular repetitive transcranial magnetic stimulation (rTMS) [e.g., 10, 11]. rTMS can enhance (high-frequency) or inhibit (low-frequency) cortical activity in targeted brain areas. It appears to increase neuroplasticity, and hence may be of value in chronic or treatment-resistant neurocircuit-based disorders, such as SE-AN [9]. Based on the Research Domain Criteria, candidate targets for rTMS in eating disorders (EDs) have been described, involving brain structures/circuitry in the cognitive control, positive and negative valences, and social processes systems [12]. Partly for theoretical reasons, but also for accessibility reasons, rTMS studies have targeted the dorsolateral prefrontal cortex (DLPFC) or the dorsomedial prefrontal cortex (DMPFC) [12].

Proof-of-concept studies have shown that rTMS is a promising treatment in AN [9, 12]. We previously carried out two single-session studies in AN [13, 14] and a case series of 20 sessions of rTMS in SE-AN [15, 11], all involving high-frequency rTMS to the left DLPFC. These studies showed that rTMS can lead to both short- and long-term improvements in ED symptoms, mood and reward-based decision making. Thus, there is a rationale for further exploring the therapeutic potential of rTMS in SE-AN.

To date, no sham-controlled RCT of rTMS in SE-AN has been conducted. The present trial (TIARA, Transcranial Magnetic Stimulation and Neuroimaging in Anorexia Nervosa) aimed to assess the feasibility of using rTMS compared to sham treatment in people with SE-AN and to inform the development of a large-scale sham-controlled RCT [16]. Our primary objective was:

a. To assess recruitment, attendance and retention rates.

Secondary objectives were:

a. To estimate the treatment effect sizes and standard deviations for outcome measures to inform future sample size calculations.

b. To determine safety and tolerability of rTMS in SE-AN.

Subsidiary objectives were to assess neuropsychological and neural correlates and predictors of rTMS treatment in AN, and to assess within-session change processes. Findings relating to these will be published elsewhere. The study rationale, aims, and tentative hypotheses, along with the trial design and methodology are described fully in a protocol paper [16].

For peer review only - http://bmjopen.bmj.com/site/about/guidelines.xhtml

METHODS

Design, participants and setting

In a double-blind parallel group, randomised control design, participants were allocated to receive 20 sessions of either real or sham high-frequency rTMS in addition to treatment-as-usual (TAU). Outcomes were assessed at baseline (pre-randomisation), post-treatment (~ 1-month post-randomisation) and at follow-up (~ 4-months post-randomisation).

Right-handed adults (≥18 years old) with a current Diagnostic and Statistical Manual of Mental Disorders (5th Edition [17]; DSM-5) diagnosis of AN and a BMI >14 kg/m^2 were eligible. Participants had to have a severe, enduring form of AN; this was defined as an illness duration of ≥3 years and completion of at least one previous course of treatment (e.g., National Institute for Health and Care Excellence ([18]; NICE)-recommended specialist psychotherapy or specialist day-patient or inpatient treatment for their ED) (We accept that there is a continuing debate on definitions of SE-AN, for review see Broomfield, et al. [19]). To take part, participants needed agreement from their ED clinician or general practitioner. Main exclusion criteria were related to contraindications to either rTMS or magnetic resonance imaging (MRI) [for details, see 16].

Participants were recruited from the Eating Disorders Unit at the South London and Maudsley NHS Foundation Trust, through online and media advertisements and through participation in other research projects. Ethical approval was given by the London - City Road & Hampstead Research Ethics Committee (REC ref: 15/LO/0196).

Potential participants underwent a screening procedure to determine eligibility [see 16 for details]. Once eligibility was determined, participants' written informed consent was obtained prior to the baseline assessment.

Randomisation and blinding

Randomisation was conducted by the King's College London (KCL) Clinical Trials Unit (CTU) using their automatic online system. Randomisation requests were submitted by study researchers via the web-based CTU system after the baseline assessment. Participants were allocated at a ratio of 1:1 to the two trial arms using a restricted stratified randomisation algorithm stratifying by prognostic factors: AN subtype (AN-restrictive or AN-binge-purge) and intensity of TAU (high: day-patient treatment, or low: outpatient or no treatment). The stratification was implemented by minimised randomisation with a random component. The first n cases (n was not disclosed) were allocated entirely at random to further enhance allocation concealment.

Participants and researchers were blinded to treatment allocation, except for one researcher who conducted follow-up assessments and unblinded participants. For practical reasons, a small proportion of rTMS sessions (116/594 sessions; 19.53%) was delivered by the unblinded researcher. All other rTMS therapists remained blinded until study data had been collected and analysed. Participants were unblinded at 4-months post-randomisation once they had completed the study. Participants who received the sham intervention were offered real rTMS (if they continued to meet eligibility criteria) after their follow-up. Assessments of blinding success were carried out for rTMS therapists and participants. For details, see supplementary information.

Intervention

Participants received 20 sessions of (real or sham) high-frequency rTMS to the left DLPFC over 20 consecutive weekdays, in addition to TAU (i.e., specialist ED outpatient or day-patient treatment, or no current treatment). Each session lasted 30 to 60 minutes, including preparation time, 20 minutes of rTMS and administration of within-session measures. rTMS sessions were conducted at the Institute of Psychiatry, Psychology & Neuroscience, KCL in a designated rTMS suite.

For peer review only - http://bmjopen.bmj.com/site/about/guidelines.xhtml

Prior to starting treatment, all participants underwent a structural MRI scan to localise the DLPFC (Talairach co-ordinates x = −45 y =45 z = 30) [20, 11] for the purpose of neuronavigation (using Brainsight™ neuronavigation software). To determine the intensity of the rTMS stimulation, a Magstim Rapid device (Magstim®, Whitland, Wales, UK) with a real TMS figure-of-eight coil was used to determine participants' motor threshold (MT), which represents membrane-related excitability of cortical axons. Using the motor-evoked potential method, the MT was established by determining the minimum stimulator output intensity required to obtain five out of ten motor-evoked potentials >50 μV. MT was acquired weekly for each participant to ensure accuracy of the rTMS dose.

The Magstim Rapid device and Magstim D70-mm air-cooled real and sham coils were used to administer real and sham rTMS. Participants in the real group received 20 sessions of high-frequency (10 Hz) rTMS at 110 % of their individual MT, consisting of twenty 5-second trains with 55-second inter-train intervals delivered to the left DLPFC (a total of 1000 pulses delivered over each 20 minute session) [11, 20]. Sham stimulation was administered at the same parameters as real rTMS; however, a sham coil was used. The sham coil produces the same noises and feelings as the real coil but does not deliver active stimulation to the brain; rather it stimulates facial and scalp nerves.

Outcomes
The primary outcomes to assess feasibility were recruitment, attendance and retention rates. To judge whether or how to proceed with a future definitive trial we pre-specified two criteria, firstly, recruitment as planned (see protocol paper [16] and the 'Changes to planned protocol' section below) and, secondly, research follow-up rates of ≥ 80% at 4-months post-randomisation. We did not pre-specify any rTMS session attendance rates required for progression to a full trial, but clearly these would also guide a decision about the feasibility of a future trial. rTMS session attendance was recorded using a specially designed case record form.

Secondary feasibility outcomes included a range of clinical measures administered at baseline, 1-month (post-treatment) and 4-months post-randomisation (follow-up) to assess ED symptomatology, mood, other psychopathology and quality of life. Neurocognitive and neuroimaging assessments of rTMS treatment (see protocol paper [16]) were also completed, but will be presented elsewhere.

ED symptomatology: BMI; Eating Disorder Examination Questionnaire (EDE-Q) version 6.0 [21], Fear of Food Measure [22], Self-Starvation Scale (SS) [23], Eating Disorder Recovery Self-Efficacy Questionnaire (EDRSQ) [24].

Measures of mood and other psychopathology: Depression, Anxiety and Stress Scale - 21 item (DASS-21) [25], Positive and Negative Affect Schedule (PANAS) [26], Profile of Mood States (POMS) [27], Revised Obsessive-Compulsive Inventory (OCI-R) [28].

Quality of life: EuroQol Quality of Life Scale (EQ-5D-5L) [29], Clinical Impairment Assessment (CIA) [30, 31].

In light of the prominent mood and quality of life component of SE-AN, and the association between these two variables in SE-AN [4], the clinical outcome to be assessed as a primary outcome in a future definitive trial would most likely be the DASS.

Additional service utilisation: Patients' additional service utilisation was assessed with a self-report version of the Clinical Service Receipt Inventory (CSRI) [32] and a specially designed case record form.

Safety, tolerability and participants' experience of treatment:

To ensure safety, participants' weight, blood pressure (sitting and standing) and pulse were monitored weekly. Routine blood tests (including full blood count, urea and electrolytes, renal and liver function tests) were conducted prior to the start of rTMS treatment and were repeated at the mid-point of

For peer review only - http://bmjopen.bmj.com/site/about/guidelines.xhtml

treatment or more frequently if clinically indicated. rTMS-associated side effects and participants' expectations and experience of treatment were also assessed (see supplementary files).

Procedure

Full details of the procedures and a table of measures-by-assessment are presented in our protocol paper [16]. All procedures were identical between groups, except for the rTMS intervention.

Baseline assessment: Participants' weight and height were measured and they completed a battery of questionnaires (described above) and neuropsychological computer tasks (not presented here). A one-hour MRI scan was also conducted. This included a structural MRI (for rTMS target localisation), functional MRI (fMRI), resting state fMRI and arterial spin labelling (not reported here). Thereafter, participants were randomly allocated to real or sham rTMS treatment.

All rTMS procedures and parameters were in accordance with the current safety and application guidelines for rTMS [33]. Treatment was delivered by researchers trained in rTMS administration.

Each rTMS session (except session 1) started with assessment of any side effects experienced since the previous session. Within-session ED cognitions were measured with VAS (relating to subsidiary aims, published separately), completed following brief cue exposure (i.e., film-clip of highly palatable foods) immediately before and after each rTMS session.

Post-treatment assessment (1-month post-randomisation): The post-treatment assessment occurred within one week of the final rTMS session and included the same elements as the baseline assessment.

Follow-up (4-months post-randomisation): This final assessment repeated the post-treatment assessment, except no MRI scan was conducted. In this session, an audio-recorded qualitative semi-structured interview was undertaken to ascertain participants' views on and experience of rTMS (published in full elsewhere) and blinding success was evaluated. Participants were then unblinded and individuals in the sham rTMS group were offered real rTMS treatment.

Changes to planned protocol

We planned to recruit 44 participants, but revised this to 30 participants because a greater than anticipated proportion of potential participants were not eligible (e.g., due to MRI/rTMS contraindications or being left-handed). These figures are in line with recommendations for feasibility trials [34] and accounted for attrition. Additionally, we removed the upper BMI limit (18.5 kg/m^2) to reflect the change in diagnostic criteria for AN in DSM-5 [17].

Patient and public involvement (PPI)

In preparation for the present study, we asked participants in our previous proof-of-concept studies on rTMS in EDs whether they would be interested in undergoing a full course of rTMS treatment, with the vast majority (41/47; 87%) answering affirmatively [35, 14]. In 2013, our early studies in rTMS were featured in a BBC TV documentary and following this approximately 50 people with AN or their relatives contacted us as they were keen to have rTMS treatment, even if they had to travel. Many people who contacted us had SE-AN, with multiple unsuccessful previous treatments. This shows that there is an unmet need in relation to treatments for SE-AN and that patients with SE-AN and their carers see rTMS as a treatment to be prioritised in research.

In planning the present study, discussions with patients/carers influenced our study design as follows: Firstly, we were originally concerned that daily rTMS treatment over 4 weeks might be too burdensome. However, our PPI advisors thought this to be acceptable. Secondly, participant feedback emphasised the importance of including a broad range of outcome measures, rather than a narrow focus on weight and eating. Thirdly, it encouraged us to include a sham control condition in the study so as to not create unfounded expectations of success that may be based on a placebo response. The completed study protocol was reviewed and enthusiastically endorsed by one person with AN who

For peer review only - http://bmjopen.bmj.com/site/about/guidelines.xhtml

had participated in our previous rTMS case series [11] and another made minor comments which were incorporated.

Participant experience of rTMS treatment and other aspects of the current study, including assessment and treatment burden, were assessed with qualitative interviews at follow-up, as briefly described above. Data will be reported elsewhere and will inform future rTMS trials in AN.

An expert by experience and a carer of a person with AN were part of our trial steering group and reviewed and advised on the conduct of the study, its dissemination and future trial design. A summary of the results of the study has been sent to all study participants and they will be provided with a copy of this article.

Data analysis

Primary feasibility outcomes are presented as n/N (%). The post-treatment and follow-up group means and standard deviations for secondary outcomes were adjusted for baseline and presented with effect sizes (Cohen's *d*) alongside 95% confidence intervals (CI). Last observation carried forward imputation was used for missing data.

RESULTS

Patient flow, attendance and retention

Patient flow is shown in the CONSORT diagram (Figure 1) and the primary feasibility outcome findings are described below. The trial duration was determined by the funding period.

During the 20-month recruitment period (August 2015-March 2017), 269 people expressed interest in the study. Of these, 61 (22.7%) did not meet the inclusion criteria and 81 (30.1%) were not interested and/or declined to participate, with the majority citing trial practicalities as a reason for this (e.g., accessibility, financial limitations, time commitment, etc.). Thirty-four people were enrolled and randomly allocated to the two treatment arms (n=17 per group). Two randomised participants were withdrawn for safety reasons prior to starting treatment: one participant (allocated to sham rTMS) had a syncope during her initial MT assessment; the other patient's (allocated to real rTMS) weight had dropped below BMI 14 kg/m^2. These participants were excluded from the analyses. All others were included.

Thirty-two participants started treatment; two participants allocated to sham rTMS stopped treatment, one after 4 sessions (due to anxiety with travel) and one after 9 sessions (due to multiple commitments). All other participants (n=30) completed treatment (defined a priori as ≥ 17 sessions of rTMS, n=18 attended the full 20 sessions) and all three study assessments, giving a retention rate of 93.75% (30/32).

Baseline demographics and clinical characteristics are presented in Table 1. All participants were female and had a long-standing illness, having previously spent a mean of nearly 11 months as an inpatient for their ED.

Table 1. Baseline demographics and clinical characteristics

	Whole sample		Real rTMS		Sham rTMS	
	N		*N*		*N*	
**Demographic details**						
Age (mean [SD])	34	29.74 (10.35)	17	28.47 (9.48)	17	31.00 (11.29)
Highest level of education achieved (*n*)	33		17		16	
GCSE		3		2		1
AS Levels and above		30		15		15
Ethnicity (*n*)	34		17		17	

For peer review only - http://bmjopen.bmj.com/site/about/guidelines.xhtml

White		31		16		15
Other		3		1		2
Marital Status (*n*)	34		17		17	
Single		26		13		13
Married		6		4		2
Divorced		1		0		1
Other		1		0		1
**Clinical characteristics**						
Diagnosis (*n*)	34		17		17	
AN-R		22		11		11
AN-BP		12		6		6
BMI, kg/m^2 (mean [SD])	33	16.00 (1.44)	17	15.76 (1.62)	16	16.26 (1.22)
Duration of illness, years (mean [SD])	34	14.07 (10.75)	17	13.74 (10.74)	17	14.41 (11.09)
Number of previous ED hospitalisations (mean [SD])	34	2.18 (1.91)	17	2.47 (2.07)	17	1.88 (1.76)
Number of previous ED inpatient stays, months (mean [SD])	33	10.49 (11.66)	17	12.37 (12.46)	16	8.50 (10.78)
Previous course (≥1) of ED outpatient treatment (*n*)	29		14		15	
Previous course (≥1) of ED day-patient treatment (*n*)	19		10		9	
Current treatment (*n*)	34		17			
Receiving ED day-patient treatment		2		1		1
Receiving ED outpatient treatment		25		13		12
Receiving no treatment		7		3		4
Antidepressant medication		21		11		10
Other psychotropic medication		7		2		5
Antipsychotic medication		3		1		3
Benzodiazepine/other anxiolytic/sedative medication		2		2		2
EDE-Q Global (mean [SD])	33	4.16 (1.11)	17	4.07 (1.28)	16	4.25 (0.94)
EDE-Q Restraint Subscale (mean [SD])	33	3.99 (1.54)	17	3.87 (1.46)	16	4.11 (1.65)
EDE-Q Eating Concern Subscale (mean [SD])	33	3.65 (1.21)	17	3.59 (1.45)	16	3.71 (0.93)
EDE-Q Shape Concern Subscale (mean [SD])	33	4.68 (1.31)	17	4.58 (1.55)	16	4.78 (1.03)
EDE-Q Weight Concern Subscale (mean [SD])	33	4.33 (1.25)	17	4.28 (1.33)	16	4.38 (1.20)
CIA Total (mean [SD])	33	43.64 (11.36)	17	43.35 (12.72)	16	43.94 (10.12)
EQ-5D-5L: How good or bad is your health today? (mean [SD])	33	48.91 (17.44)	17	47.47 (18.63)	16	50.44 (16.55)
DASS-21 Depression (mean [SD])	33	26.12 (9.68)	17	26.82 (9.44)	16	25.38 (10.19)
DASS-21 Anxiety (mean [SD])	33	15.39 (10.29)	17	14.82 (8.31)	16	16.00 (12.31)
DASS-21 Stress (mean [SD])	33	26.91 (7.92)	17	28.35 (7.12)	16	25.38 (8.66)
DASS-21 Total (mean [SD])	33	68.42 (24.52)	17	70.00 (20.59)	16	66.75 (28.72)
POMS Total Mood Disturbance (mean [SD])	33	83.97 (36.75)	17	81.41 (36.84)	16	86.69 (37.66)
OCI-R Total (mean [SD])	33	27.79 (16.97)	17	24.00 (16.48)	16	31.81 (17.05)

rTMS = repetitive transcranial magnetic stimulation; AN-R = anorexia nervosa restrictive subtype; AN-BP = anorexia nervosa binge-purge subtype; ED = eating disorder; EDE-Q = Eating Disorder Examination Questionnaire; DASS= Depression, Anxiety and Stress Scale; CIA = Clinical Impairment Assessment; EQ-5D-5L = EuroQol Quality of Life Scale; OCI-R = Revised Obsessive-Compulsive Inventory

For peer review only - http://bmjopen.bmj.com/site/about/guidelines.xhtml

Treatment effect sizes

The means, standard deviations and between-group treatment effect sizes (with confidence intervals) for change scores (baseline to post-treatment and baseline to follow-up) of the secondary clinical outcomes are presented in Table 2. Group differences in BMI and ED symptoms were of small effect at both post-treatment and follow-up, but favoured active treatment. At 4-months post-randomisation, there were between-group differences of medium to large effect size in measures of mood, obsessive compulsive symptoms and quality of life, all favouring the active treatment. The adjusted means for the planned future primary outcome, DASS total score, were -21.25 (SD 24.33) in the real intervention group and -3.75 (SD 12.75) in the sham group, with a between-group effect size of *d*= -0.9 (95% CI -1.62 to -0.17).

For peer review only

For peer review only - http://bmjopen.bmj.com/site/about/guidelines.xhtml

Table 2. The mean change scores (post-treatment and follow-up scores adjusted for baseline) for the secondary clinical outcome measures, including the number of participants included in the analysis (N), means, and standard deviations (SD) for each group, and the estimated effect size (Cohen's *d*) with 95% confidence intervals (95% CI).

	Post-treatment (adjusted for baseline)							Follow-up (adjusted for baseline)						
	Real			Sham			*d* (95% CI)	Real			Sham			*d* (95% CI)
**Assessment**	*N*	Mean	SD	*N*	Mean	SD		*N*	Mean	SD	*N*	Mean	SD	
**ED-related outcomes**														
BMI	16	0.11	0.73	16	-0.08	0.32	*0.33 (-0.37, 1.03)*	16	0.28	1.25	16	0.04	1.05	*0.2 (-0.49, 0.90)*
EDE-Q Global	16	-0.28	0.73	16	-0.4	0.79	*0.16 (-0.54, 0.85)*	16	-0.43	0.83	16	-0.52	0.87	*0.1 (-0.60, 0.79)*
Self-Starvation Scale	16	-4	16.15	16	-6.81	13.53	*0.19 (-0.51, 0.88)*	16	-13.06	20.78	16	-9.6	13.68	*-0.2 (-0.89, 0.50)*
FoFM Anxiety About Eating	16	-2.94	6.35	16	-4.44	8.97	*0.19 (-0.50, 0.89)*	16	-4.69	6.39	16	-4.56	8.74	*-0.02 (-0.71, 0.68)*
FoFM Food Avoidance Behaviours	16	-3.56	5.77	16	-3	3.39	*-0.12 (-0.81, 0.58)*	16	-3.5	6	16	-1.69	5.51	*-0.32 (-1.01, 0.39)*
FoFM Feared Concerns	16	-2	6.5	16	-1.81	9.74	*-0.02 (-0.72, 0.67)*	16	-2.63	6.79	16	-1.5	9.25	*-0.14 (-0.83, 0.56)*
EDRSQ Normative Eating Self-Efficacy	16	0	0.56	16	0.13	0.62	*-0.22 (-0.92, 0.47)*	16	0.26	0.77	16	0.29	0.59	*-0.04 (-0.73, 0.66)*
EDRSQ Body Image Self-Efficacy	16	-0.08	0.49	16	-0.11	0.61	*0.06 (-0.63, 0.75)*	16	0.08	0.47	16	0.16	0.61	*-0.14 (-0.83, 0.56)*
**Clinical impairments/quality of life**														
CIA	16	-6.31	12.37	16	-4.69	5.87	*-0.17 (-0.86, 0.53)*	16	-9.56	15.66	16	-6	9.19	*-0.28 (-0.97, 0.42)*

For peer review only - http://bmjopen.bmj.com/site/about/guidelines.xhtml

For peer review only

EQ-5D-5L: How good or bad is your health today?	8	-0.25	19.65	10	7.7	16.67	*-0.44 (-1.38, 0.51)*	16	13.06	18.31	16	4.81	13.15	*0.52 (-0.19, 1.22)*
Mood/affect/anxiety														
DASS-21 Depression	16	-5.13	8.94	16	-3.25	10.55	*-0.19 (-0.89, 0.51)*	16	-9.13	10.61	16	-1.13	8.58	***-0.83 (-1.55, -0.10)***
DASS-21 Anxiety	16	-7.25	6.15	16	-4.13	5.44	*-0.54 (-1.24, 0.17)*	16	-4.88	7.19	16	-1	4.79	*-0.63 (-1.34, 0.08)*
DASS-21 Stress	16	-6.75	9.26	16	-4.5	4.82	*-0.31 (-1.00, 0.40)*	16	-7.25	9.71	16	-1.63	3.88	***-0.76 (-1.47, -0.04)***
DASS-21 Total	16	-19.13	21.8	16	-11.88	17.73	*-0.37 (-1.06, 0.34)*	16	-21.25	24.33	16	-3.75	12.75	***-0.9 (-1.62, -0.17)***
PANAS Positive Affect	16	1.75	5.23	16	1.06	5.4	*0.13 (-0.57, 0.82)*	16	4.56	5.79	16	0.13	3.88	*0.9 (0.17, 1.62)*
PANAS Negative Affect	16	-3.81	9.4	16	-1.44	5.63	*-0.31 (-1.00, 0.39)*	16	-7	9.13	16	-1.94	7.42	*-0.61 (-1.31, 0.11)*
POMS Total Mood Disturbance	16	-9.88	37.68	16	-8.06	21.2	*-0.06 (-0.75, 0.63)*	16	-36.75	39.08	16	-5.5	20.82	***-1 (-1.73, -0.25)***
OCI-R Total	16	-3.69	7.55	16	0.94	5.58	*-0.7 (-1.41, 0.02)*	16	-1.88	8.13	16	0.81	7.84	*-0.34 (-1.03, 0.36)*

Bold font signifies that the confidence intervals do not include 0.

N = number; SD = standard deviation; *d* = Cohen's *d*; 95% CI = 95% confidence intervals; BMI = body mass index; EDE-Q = Eating Disorder Examination Questionnaire; FoFM = Fear of Food Measure; EDRSQ = Eating Disorder Recovery Self-Efficacy Questionnaire; EQ-5D-5L = EuroQol Quality of Life Scale; CIA = Clinical Impairment Assessment; DASS-21 = Depression, Anxiety and Stress Scale - 21 item; PANAS = Positive and Negative Affect Schedule; POMS = Profile of Mood States; OCI-R = Revised Obsessive-Compulsive Inventory

For peer review only

For peer review only - http://bmjopen.bmj.com/site/about/guidelines.xhtml

Additional service utilisation

At baseline, twenty-five participants received outpatient treatment and five were not receiving treatment. One participant per group received day-patient treatment. A high proportion of participants were taking antidepressants and remained on this at a stable dose throughout the trial. Participants' utilisation of TAU is shown in Table 1.

At follow-up, the two participants originally in day-patient treatment were instead receiving outpatient treatment. Three participants had increased treatment intensity at follow-up, with two (one per group) starting inpatient treatment and one (from the real group) starting day-patient treatment. Of the remaining participants, two initially receiving no treatment started outpatient treatment and eight decreased intensity from outpatient treatment to no treatment.

Of those who completed the sham intervention, 71% took up the offer of having real rTMS treatment.

Safety

In addition to the one withdrawn participant whose weight dropped below range prior to starting treatment, one other participant's weight (from the real group) was recorded below BMI 14kg/m^2 (13.80 kg/m^2) in their final rTMS session. No other participants' weight fell below the required BMI range for the duration of treatment. Blood pressure and pulse measurements did not raise any undue concerns during the study. One participant had lowered baseline potassium and start of treatment was delayed by one week. Blood samples for the remaining participants raised no major concerns, i.e., termination or postponing of treatment was not required. For side effects experienced, see Supplementary Table 1.

DISCUSSION

Principal findings

The main findings relate to the primary feasibility objectives of this study. We were able to recruit participants as planned, after making an adjustment to recruitment numbers. Many people interested in the trial could not be recruited as travelling to London for rTMS sessions proved impractical. A future trial therefore needs to consider offering treatment in several centres with easy transport access. Research follow-up rates exceeded our pre-specified criterion of $\geq$ 80%. Treatment session attendance was excellent in both groups. Although for pragmatic reasons, and compared to others, our definition of 'severe, enduring AN' was lenient [19], we managed to recruit and retain a very chronic and treatment-refractory population.

In relation to our secondary feasibility objectives, there were large between-group effect sizes on change scores from pre-treatment to follow-up on several mood variables (e.g., DASS global score d=-0.9, -1.62 to -0.17), favouring real rTMS. Comorbid depression is common in AN and has been shown to be associated with poor quality of life in people with SE-AN [4]. The importance of improving quality of life in SE-AN, rather than focussing on changing ED symptoms and weight gain has been emphasised [36], and the improvements in depression observed here may contribute to the broader aim of enhancing quality of life in this group. Also, given that antidepressants are typically not very effective in underweight populations or have unacceptable side effects [1], rTMS may provide an alternative treatment for common comorbid symptoms such as depression and anxiety. Within the current study, a higher proportion of participants were taking antidepressant medication (61.7%; 21/34 participants) and somewhat higher depression scores were observed, compared to other treatment studies of AN [37, 38]. This may suggest that either our participants had particularly high levels of comorbid depression or that we attracted participants who were particularly drawn to 'physical/biologically-targeted treatments' rather than psychological treatments. Having said that, many participants had previously undertaken unsuccessful psychological treatments.

We considered that rTMS may be interacting with the actions of the medication to produce this antidepressant effect, however, there is no evidence for this mechanism in the depression literature.

For peer review only - http://bmjopen.bmj.com/site/about/guidelines.xhtml

Developing better evidence for the treatment of comorbidities in EDs is a research recommendation in the recent NICE guidelines [18] and, therefore, our study potentially fills an important gap.

In addition to the mood effects, there were medium between-group effect sizes on follow-up change scores in quality of life (d=0.52, 95% CI -0.19-1.22), whereas between-group effect sizes on change scores for BMI (d=0.2, 95% CI -0.49-0.90) and ED symptoms (d=0.1, 95% CI -0.60-0.79) were small. Larger between-group effect sizes were seen on change scores from pre-treatment to follow-up than to post-treatment, suggesting that changes develop over time, rather than being due to immediate effects of rTMS. A similar delay in effect was observed in our previous case series of rTMS in SE-AN [11].

rTMS was safe, well tolerated and considered to be an acceptable treatment by participants. These various findings suggest that it is feasible to conduct a future larger-scale therapeutic RCT with a sham-controlled design to establish the therapeutic efficacy of rTMS in SE-AN.

Strengths and limitations

Our study has several strengths. It is the first RCT of multi-session rTMS treatment in individuals with AN. Secondly, it focused on people with severe, enduring illness. As such, it adds to the limited number of studies that have specifically targeted people with SE-AN. Thirdly, it was sham-controlled, which is considered the gold-standard method of evaluating the clinical efficacy of rTMS treatment [39]. Fourthly, the majority of participants did not correctly guess their treatment allocation at follow-up, suggesting blinding was successful (see Supplementary Figure 1 and 2). Lastly, the rTMS was individualised through the use of neuronavigation and a wide range of measures to assess relevant clinical outcomes were used.

In terms of limitations, the duration of the follow-up period was relatively short [11]. Our choices regarding the rTMS protocol and target brain area (left DLPFC) were theoretically-, evidentially- and practically-based [12]; however, the optimal brain areas to target and the rTMS protocols to administer in SE-AN are unknown. We used a shorter illness duration (minimum of 3 years) than what is commonly used to define SE-AN (e.g. 7 years [19]), but nonetheless managed to recruit participants with a long-standing illness who had typically received several previous courses of intensive treatment. Our attempts to keep researchers blind to treatment allocation were only partly successful; approximately 20% of rTMS sessions were delivered by an unblinded researcher, and another researcher correctly guessed treatment allocation of participants.

Strengths and limitations in relation to other studies

Research into treatments for people with SE-AN is limited [36]. In addition to this study, there have only been two trials with a focus on SE-AN. The first of these assessed the efficacy of 30 sessions of cognitive behavioural therapy for AN compared to specialist supportive clinical management in 63 patients [6]. Between-group differences in clinical outcomes were minimal. Within-group assessments showed small to moderate effect sizes for BMI-change and medium to large for ED symptoms, depression and quality of life from baseline to end of treatment and to 6- and 12-month follow-up. The second study investigated the effects of four weeks of a synthetic cannabinoid agonist (dronabinol) versus placebo as an adjunct to a multi-modal treatment combining psychotherapy with nutritional interventions in 25 patients with SE-AN [7]. Dronabinol produced significantly greater short-term weight gain than placebo, but changes in ED symptoms were minimal during the study period. No follow-up data were reported. In both of these studies, treatment drop-out rates were low, as in the current study, highlighting the desire of people with SE-AN to participate in novel treatments.

Implications for future research

Building on the present study, a large-scale multi-centre RCT of real versus sham rTMS as an adjunct to TAU with a similar design, should be considered. Such a trial should include a longer follow-up

For peer review only - http://bmjopen.bmj.com/site/about/guidelines.xhtml

period (e.g., 6- and 12-months) to assess the persistence or otherwise of rTMS effects. This is also of importance given that neuroplastic changes develop over time [40]. For example, studies of deep brain stimulation in AN have shown that in treatment responders, changes in mood predate those in ED symptoms by several months [e.g., 41]. Relatedly, it would be desirable to include multimodal assessment of comorbidities, for example, using a combination of semi-structured interviews, observed-rated measures and self-reports. Secondly, an assessment of the cost-effectiveness of establishing rTMS as a treatment option for SE-AN should be carried out. Inclusion of inpatients with SE-AN in a future trial would be desirable, as it would be easier for them to attend daily sessions. This might also allow inclusion of patients with a BMI <14 kg/m^2, given that inpatients have regular medical monitoring and that their food intake is more regular than that of community-dwelling patients.

Several questions need to be considered in future research of rTMS in SE-AN. The optimal brain areas to target and the rTMS protocols for SE-AN are not known. High-frequency rTMS targeting the DLPFC was chosen for the current study as it was hypothesised that this would remediate the hypoactivity observed in AN in response to symptom provocation, cognitive flexibility and set-shifting tasks, and thus re-balance cognitive control and reward systems [12]. It was also selected given the strong evidence base for high-frequency DLPFC rTMS in other neurocircuit-based disorders (e.g., treatment-resistant depression [42]). Following on from research in depression, the use of low-frequency rTMS or intermittent theta burst stimulation (iTBS) in comparison to high-frequency rTMS might be tested. Low-frequency rTMS is thought to have fewer side effects and be more well-tolerated, and iTBS would substantially reduce treatment time and participant burden. In the depression literature, it appear that both of these have similar levels of efficacy to high-frequency rTMS [43-46]. Future studies should also consider rTMS as an adjunct to psychological therapies [47]. Other neuromodulation treatments in combination with cognitive interventions have shown promise [48, 49], and so, addition of rTMS to structured psychotherapy or cognitive training tasks in SE-AN may help increase its efficacy [12]. Finally, additional work on neural and neurocognitive mechanisms of action of rTMS and the cost-effectiveness of this treatment are necessary.

CONCLUSION

In this feasibility RCT, rTMS was safe and well tolerated. This study provides preliminary evidence for the therapeutic potential of rTMS treatment in community-dwelling SE-AN as an adjunct to TAU. It suggests that it is feasible to conduct a future larger-scale therapeutic RCT with a sham-controlled design to establish/confirm the therapeutic efficacy of rTMS in AN. The findings from this trial will inform a future large-scale RCT with respect to decisions on primary outcome measures and other aspects of protocol development, such as sample size, design, location and number of research centres. Future studies should include a longer follow-up period and a formal assessment of cost-effectiveness. Consideration should also be given to use of alternative stimulation protocols (e.g., low-frequency rTMS) and the combination of rTMS and ED-specific therapies/tasks to maximise impact upon ED and mood.

For peer review only

For peer review only - http://bmjopen.bmj.com/site/about/guidelines.xhtml

ACKNOWLEDGEMENTS
We would like to thank the individuals who participated in this study for their time and commitment and our PPI advisors for their valuable feedback. The authors would also like to thank Daniela Mercado Beivide and Pia Lauffer for their assistance in data collection, the radiographers at the KCL Centre for Neuroimaging Sciences for their work during with the MRI scans and their guidance on decisions regarding eligibility and protocol development, and the phlebotomy staff at South London and Maudsley Outpatients'. This study was supported by the United Kingdom Clinical Research Collaboration-registered King's Clinical Trials Unit at King's Health Partners, which is part funded by the NIHR Biomedical Research Centre for Mental Health at South London and Maudsley NHS Foundation Trust and King's College London and the NIHR Evaluation, Trials and Studies Coordinating Centre.

CONTRIBUTORS

BD, SB, JM and US drafted the manuscript. BD and SB conducted data analysis, which was supervised by BC and US. OOD, ICC, MK, SJR, NK, DG and ASD revised the manuscript critically for important intellectual content. Ethical approval was obtained by SB, JM, MK and US. Funding from NIHR was obtained by US, JM and ICC. Funding from the NIHR BRC was obtained by SB, JM, MK, OOD, ICC and US. JM, SB and US registered the trial on the ISRCTN registry. JM, SB, MK, US and BD were involved in participant recruitment. BD, SB, JM, MK, JW and SJR were involved in data collection. rTMS treatment was provided by BD, SB, JM and MK. OOD, MK, JW, SJR, SB, JM, ICC and US contributed to the design and conception of the study. All authors, were involved in drafting, critiquing and approving of the manuscript, and accept responsibility for the accuracy and integrity of this work.

Protocol
The study protocol was published prior to recruitment [16].

Funding
This work was supported by a National Institute for Health Research (NIHR) Research for Patient Benefit (RfPB) grant (RB-PG-1013-32049) and Infrastructure Support for Pilot studies from the NIHR Biomedical Research Centre (BRC) at South London and Maudsley NHS Foundation Trust (SLaM) and King's College London (KCL). This paper presents independent research funded by the National Institute for Health Research (NIHR) under its Research for Patient Benefit (RfPB) Programme (Grant Reference Number PB-PG-1013-32049). Owen O'Daly receives salary support from an NIHR Infrastructure grant for the Wellcome Trust/KCL Clinical Research Facility. Ulrike Schmidt is supported by an NIHR Senior Investigator Award. Ulrike Schmidt, Anthony David and Iain Campbell receive salary support from the NIHR Mental Health BRC at SLaM NHS Foundation Trust and KCL. The views expressed are those of the author(s) and not necessarily those of the NHS, the NIHR or the Department of Health.

Competing Interests
None declared.

Data sharing statement
No additional data are available.

For peer review only - http://bmjopen.bmj.com/site/about/guidelines.xhtml

REFERENCES

1. Zipfel S, Giel KE, Bulik CM, et al. Anorexia nervosa: aetiology, assessment, and treatment. *Lancet Psychiatry* 2015;2:1099-111.
2. Treasure J, Zipfel S, Micali N, et al. Anorexia nervosa. *Nat Rev Dis Primers* 2015;1:15074.
3. Steinhausen HC. The outcome of anorexia nervosa in the 20th century. *Am J Psychiatry* 2002;159:1284-93.
4. Arkell J, Robinson P. A pilot case series using qualitative and quantitative methods: biological, psychological and social outcome in severe and enduring eating disorder (anorexia nervosa). *Int J Eat Disord* 2008;41:650-6.
5. Brockmeyer T, Friederich HC, Schmidt U. Advances in the treatment of anorexia nervosa: a review of established and emerging interventions. *Psychol Med* 2017:1-37.
6. Touyz S, Le Grange D, Lacey H, et al. Treating severe and enduring anorexia nervosa: a randomized controlled trial. *Psychol Med* 2013;43:2501-11.
7. Andries A, Frystyk J, Flyvbjerg A, et al. Dronabinol in severe, enduring anorexia nervosa: a randomized controlled trial. *Int J Eat Disord* 2014;47:18-23.
8. Hay PJ, Touyz S, Sud R. Treatment for severe and enduring anorexia nervosa: a review. *Aust N Z J Psychiatry* 2012;46:1136-44.
9. Dalton B, Campbell IC, Schmidt U. Neuromodulation and neurofeedback treatments in eating disorders and obesity. *Curr Opin Psychiatry* 2017;30:458-73.
10. Choudhary P, Roy P, Kumar Kar S. Improvement of weight and attitude towards eating behaviour with high frequency rTMS augmentation in anorexia nervosa. *Asian J Psychiatr* 2017;28:160.
11. McClelland J, Kekic M, Campbell IC, et al. Repetitive transcranial magnetic stimulation (rTMS) treatment in enduring anorexia nervosa: a case series. *Eur Eat Disord Rev* 2016;24:157-63.
12. Dunlop KA, Woodside B, Downar J. Targeting neural endophenotypes of eating disorders with non-invasive brain stimulation. *Front Neurosci* 2016;10:30.
13. McClelland J, Kekic M, Bozhilova N, et al. A randomised controlled trial of neuronavigated repetitive transcranial magnetic stimulation (rTMS) in anorexia nervosa. *PLOS One* 2016;11:e0148606.
14. Van den Eynde F, Guillaume S, Broadbent H, et al. Repetitive transcranial magnetic stimulation in anorexia nervosa: a pilot study. *Eur Psychiatry* 2013;28:98-101.
15. McClelland J, Bozhilova N, Nestler S, et al. Improvements in symptoms following neuronavigated repetitive transcranial magnetic stimulation (rTMS) in severe and enduring anorexia nervosa: findings from two case studies. *Eur Eat Disord Rev* 2013;21:500-6.
16. Bartholdy S, McClelland J, Kekic M, et al. Clinical outcomes and neural correlates of 20 sessions of repetitive transcranial magnetic stimulation in severe and enduring anorexia nervosa (the TIARA study): study protocol for a randomised controlled feasibility trial. *Trials* 2015;16:548.
17. American Psychiatric Association. Diagnostic and Statistical Manual of Mental Disorders (5th ed.). Arlington, VA: American Psychiatric Publishing, 2013.
18. National Institute for Health and Care Excellence. Eating disorders: recognition and treatment. NICE Clinical Guideline NG69. 2017. https://www.nice.org.uk/guidance/ng69 (accessed 27th November 2017).
19. Broomfield C, Stedal K, Touyz S, et al. Labeling and defining severe and enduring anorexia nervosa: a systematic review and critical analysis. *Int J Eat Disord* 2017;50:611-23.
20. Fitzgerald PB, Hoy K, McQueen S, et al. A randomized trial of rTMS targeted with MRI based neuro-navigation in treatment-resistant depression. *Neuropsychopharmacology* 2009;34:1255-62.
21. Fairburn C. Appendix: Eating Disorder Examination Questionnaire (EDE-Q Version 6.0) Cognitive Behaviour Therapy and Eating Disorders. New York: Guilford Press, 2008.
22. Levinson CA, Byrne M. The fear of food measure: a novel measure for use in exposure therapy for eating disorders. *Int J Eat Disord* 2015;48:271-83.
23. Godier LR, Park RJ. A novel measure of compulsive food restriction in anorexia nervosa: validation of the Self-Starvation Scale (SS). *Eat Behav* 2015;17:10-13.

For peer review only - http://bmjopen.bmj.com/site/about/guidelines.xhtml

24. Marinilli Pinto A, Guarda AS, Heinberg LJ, et al. Development of the eating disorder recovery self-efficacy questionnaire. *Int J Eat Disord* 2006;39:376-84.
25. Lovibond S, Lovibond P. Manual for the Depression Anxiety Stress Scales. 2nd ed. Sydney: Psychology Foundation, 1995.
26. Watson D, Clark LA, Tellegen A. Development and validation of brief measures of positive and negative affect: the PANAS scales. *J Pers Soc Psychol* 1988;54:1063-70.
27. McNair D, Lorr M, Droppleman L. Profile of Mood States Manual. San Diego: Educational and Industrial Testing Services, 1992.
28. Foa EB, Huppert JD, Leiberg S, et al. The Obsessive-Compulsive Inventory: development and validation of a short version. *Psychol Assess* 2002;14:485-96.
29. Herdman M, Gudex C, Lloyd A, et al. Development and preliminary testing of the new five-level version of EQ-5D (EQ-5D-5L). *Qual Life Res* 2011;20:1727-36.
30. Bohn K, Doll HA, Cooper Z, et al. The measurement of impairment due to eating disorder psychopathology. *Behav Res Ther* 2008;46:1105-10.
31. Bohn K, Fairburn CG. The Clinical Impairment Assessment questionnaire (CIA). Cognitive Behavioral Therapy for Eating Disorders. New York: Guilford Press, 2008.
32. Beecham J, Knapp M. Costing psychiatric interventions. In: Thornicroft G, ed. Measuring Mental Health Needs. London: Gaskell, 2001:200-24.
33. Rossi S, Hallett M, Rossini PM, et al. Safety, ethical considerations, and application guidelines for the use of transcranial magnetic stimulation in clinical practice and research. *Clin Neurophysiol* 2009;120:2008-39.
34. Lancaster GA, Dodd S, Williamson PR. Design and analysis of pilot studies: recommendations for good practice. *J Eval Clin Pract* 2004;10:307-12.
35. Van den Eynde F, Claudino AM, Mogg A, et al. Repetitive transcranial magnetic stimulation reduces cue-induced food craving in bulimic disorders. *Biological psychiatry* 2010;67:793-5.
36. Wonderlich S, Mitchell JE, Crosby RD, et al. Minimizing and treating chronicity in the eating disorders: a clinical overview. *Int J Eat Disord* 2012;45:467-75.
37. Schmidt U, Magill N, Renwick B, et al. The Maudsley Outpatient Study of Treatments for Anorexia Nervosa and Related Conditions (MOSAIC): comparison of the Maudsley Model of Anorexia Nervosa Treatment for Adults (MANTRA) with specialist supportive clinical management (SSCM) in outpatients with broadly defined anorexia nervosa: a randomized controlled trial. *J Consult Clinl Psychol* 2015;83:796.
38. Byrne S, Wade T, Hay P, et al. A randomised controlled trial of three psychological treatments for anorexia nervosa. *Psychol Med* 2017:1-11.
39. Gaynes BN, Lloyd SW, Lux L, et al. Repetitive transcranial magnetic stimulation for treatment-resistant depression: a systematic review and meta-analysis. *J Clin Psychiatry* 2014;75:477-89; quiz 89.
40. Hallett M. Transcranial magnetic stimulation and the human brain. *Nature* 2000;406:147-50.
41. Lipsman N, Lam E, Volpini M, et al. Deep brain stimulation of the subcallosal cingulate for treatment-refractory anorexia nervosa: 1 year follow-up of an open-label trial. *Lancet Psychiatry* 2017;4:285-94.
42. Leggett LE, Soril LJ, Coward S, et al. Repetitive Transcranial Magnetic Stimulation for Treatment-Resistant Depression in Adult and Youth Populations: a Systematic Literature Review and Meta-Analysis. *Prim Care Companion CNS Disord* 2015;17.
43. Brunelin J, Jalenques I, Trojak B, et al. The efficacy and safety of low frequency repetitive transcranial magnetic stimulation for treatment-resistant depression: the results from a large multicenter French RCT. *Brain Stimul* 2014;7:855-63.
44. Fitzgerald PB, Brown TL, Marston NA, et al. Transcranial magnetic stimulation in the treatment of depression: a double-blind, placebo-controlled trial. *Arch Gen Psychiatry* 2003;60:1002-8.
45. Bakker N, Shahab S, Giacobbe P, et al. rTMS of the dorsomedial prefrontal cortex for major depression: safety, tolerability, effectiveness, and outcome predictors for 10 Hz versus intermittent theta-burst stimulation. *Brain Stimul* 2015;8:208-15.
46. Dunlop K, Gaprielian P, Blumberger D, et al. MRI-guided dmPFC-rTMS as a Treatment for Treatment-resistant Major Depressive Disorder. *J Vis Exp* 2015:e53129.

For peer review only - http://bmjopen.bmj.com/site/about/guidelines.xhtml

47. Bajbouj M, Padberg F. A perfect match: noninvasive brain stimulation and psychotherapy. *Eur Arch Psychiatry Clin Neurosci* 2014;264 Suppl 1:S27-33.
48. De Raedt R, Vanderhasselt MA, Baeken C. Neurostimulation as an intervention for treatment resistant depression: from research on mechanisms towards targeted neurocognitive strategies. *Clin Psychol Rev* 2015;41:61-9.
49. Elmasry J, Loo C, Martin D. A systematic review of transcranial electrical stimulation combined with cognitive training. *Restor Neurol Neurosci* 2015;33:263-78.

FIGURES

Figure 1. Consort diagram of participant involvement.

Supplementary File, Figure 1. Blinding guesses combined with level of certainty for all participants in the real rTMS intervention group.

Supplementary File, Figure 2. Blinding guesses combined with level of certainty for all participants in the sham rTMS intervention group.

For peer review only

For peer review only - http://bmjopen.bmj.com/site/about/guidelines.xhtml

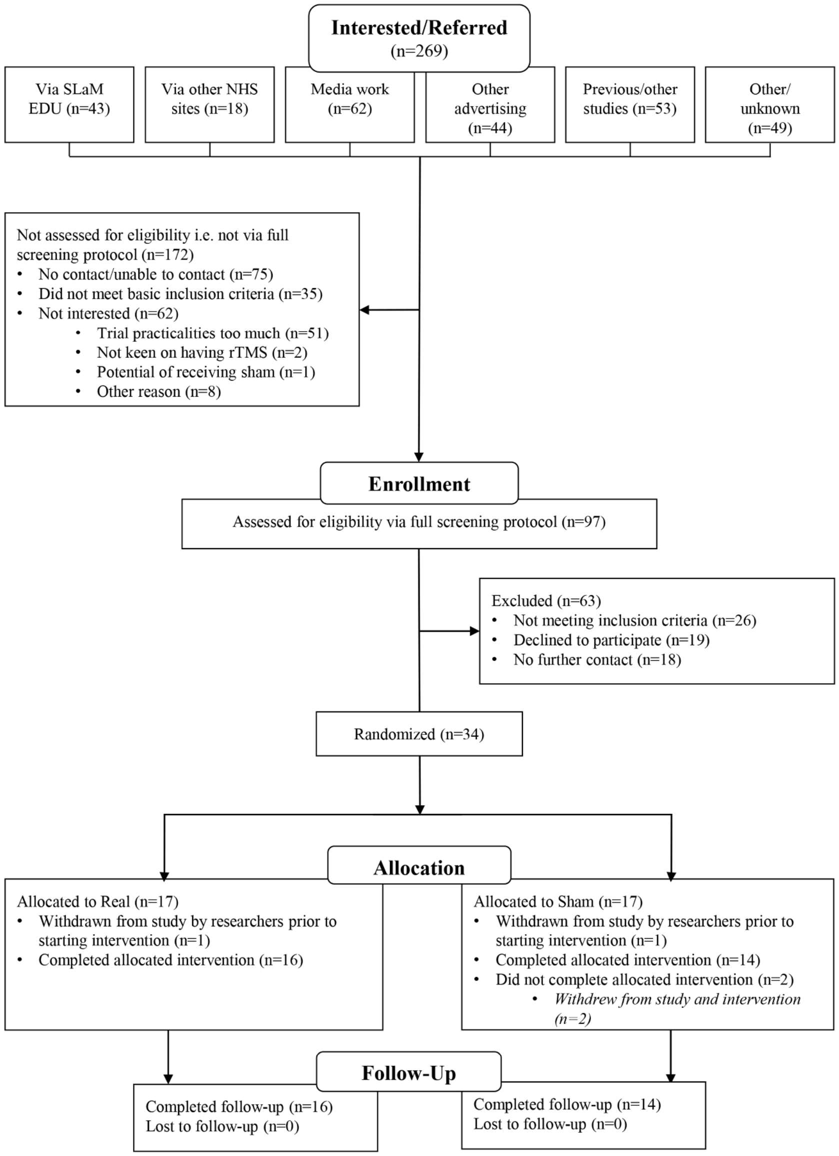

Figure 1. Consort diagram of participant involvement.

234x330mm (300 x 300 DPI)

A randomised controlled feasibility trial of real versus sham repetitive transcranial magnetic stimulation treatment in adults with severe and enduring anorexia nervosa: the TIARA study.

Dalton, Bartholdy, McClelland et al.

SUPPLEMENTARY METHODS

Blinding

Allocation concealment:

Unblinded treatment arm allocations were sent to a member of research staff who was independent from the trial team and had no contact with participants. Prior to trial commencement, this researcher labelled the real and sham rTMS coils with a coloured (red/green) keyring to blind the rTMS therapists to treatment allocation. This researcher then converted the unblinded randomisation allocation into the blinded treatment codes (red/green), and passed on this blinded allocation information to the rTMS therapists.

Assessment of blinding success:

Following a participant's rTMS treatment (at approximately 1 month post-randomisation), all researchers involved in their treatment provided a guess as to the participant's treatment allocation, and indicated certainty using a 10cm visual analogue (VAS) scale.

Every 5 sessions, participants were asked to guess their treatment allocation and record their certainty in their guess using a 10cm VAS scale.

Safety, tolerability and participants' experience of treatment

Assessment of side-effects:

rTMS-associated side effects were assessed at the beginning of each session (except session 1), with participants reporting on a 10cm visual analogue scale (VAS) whether they had experienced any of the following common rTMS side effects: headache, discomfort on the head, nausea, dizziness, light headedness, skin irritation or drowsiness. Participants were also able to record any other side effects they experienced.

Participants' expectations and experience of treatment:

Semi-structured qualitative interviews assessing treatment expectations and experience were conducted at follow-up (4-months post-randomisation). Findings will be reported in full elsewhere. A VAS of participants' expectations regarding the effects of rTMS on their condition (0 = 'I expect to feel much worse' and 10 = 'I expect to feel much better') was carried out in their first rTMS session.

SUPPLEMENTARY RESULTS

Blinding

Participant blinding:

Profile plots of participants' blinding guess combined with their rating of certainty are presented below separately for the real (see Figure 1) and sham (see Figure 2) intervention group. Each line in the profile plot refers to a different participant, the thick black line provides the median. A data point in the top part of the graph reflects that the participant guessed real. A data point in the bottom part of the graph reflects that the participant guessed sham. Certainty scores are reflected on the scale of 0 - 10/-10, with 0 meaning that the participant was completely unsure of their blinding decision and 10/10 meaning the participant was completely sure of their blinding decision.

For peer review only - http://bmjopen.bmj.com/site/about/guidelines.xhtml

A randomised controlled feasibility trial of real versus sham repetitive transcranial magnetic stimulation treatment in adults with severe and enduring anorexia nervosa: the TIARA study.

Dalton, Bartholdy, McClelland et al.

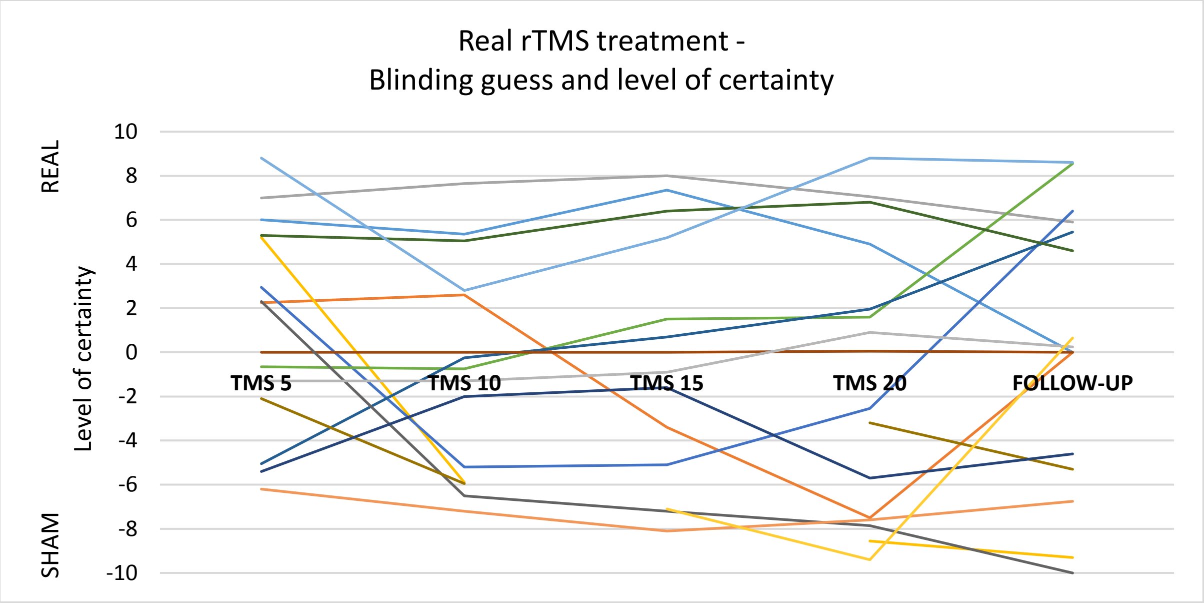

Figure 1. Blinding guesses combined with level of certainty for all participants in the real rTMS intervention group.

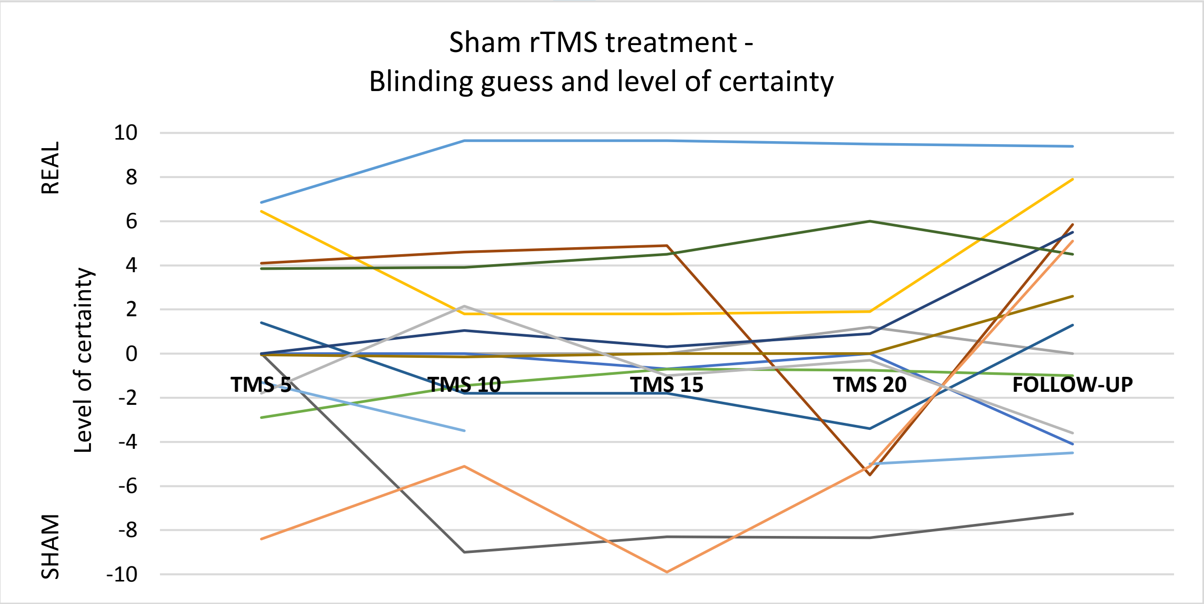

Figure 2. Blinding guesses combined with level of certainty for all participants in the sham rTMS intervention group.

Researcher blinding:

The rTMS therapists provided an allocation guess for each participant that they had administered rTMS to, once the participant had completed treatment. JM provided blinding guesses on n=17, correctly guessing the allocation of 41% of these participants. SB provided blinding guesses on n=29, correctly guessing the allocation of 31% of these participants. MK providing blinding guesses on

n=26, correctly guessing the allocation of 100% of these participants. Generally, certainty in their blinding guesses increased as the trial progressed.

Safety, tolerability, side effects and participants' expectations and experience of treatment

Tolerability and side effects:
Mean frequency and maximum intensity/severity of reported side effects are presented in Supplementary Table 1. In both groups, headaches were the most frequently reported side effect, followed by drowsiness in the real group and nausea in the sham group. Generally, the intensity/severity of side effects reduced as rTMS sessions progressed. Mean frequency of side effects was slightly higher in the real group compared to the sham group; however, the sham group experienced the same range of side effects and some had greater reported intensity/severity (e.g., headaches, nausea, dizziness, light-headedness). One participant reported chest pains as a side effect. Sessions were postponed and resumed after two days once the participant was medically cleared.

Treatment expectations:
The mean rating of participants' expectation of the efficacy of rTMS sessions in improving their condition was 6.63 (SD 1.78, range 1.8-10), with the majority of participants (n=27) expecting some clinical improvement (rating of 5-10).

Treatment experience:
Preliminary analysis of the qualitative interviews (full analyses of these data will be presented elsewhere; NB 29 transcripts available, *n*=1 transcript missing due to technical problems with recording) of patient treatment experiences suggests that many participants found attending the rTMS clinic a "very positive" and "really interesting" experience and they "enjoyed the sessions" (22/29 participants). Several participants (14/29) highlighted practical difficulties and the commitment involved: "it was quite hard travelling up every day", "it would be easier if you didn't have to do it every day", "took a lot of time and commitment"; however, some found it "much easier" than they thought it would be once they "got used to the routine" (7/29). Participants (18/29) also reported that whilst rTMS initially felt uncomfortable due to the tapping sensation on the head, any discomfort experienced lessened over time as they "got used to it".

For peer review only - http://bmjopen.bmj.com/site/about/guidelines.xhtml

A randomised controlled feasibility trial of real versus sham repetitive transcranial magnetic stimulation treatment in adults with severe and enduring anorexia nervosa: the TIARA study.

Dalton, Bartholdy, McClelland et al.

	Real					Sham				
**Side effects**	*Total*	Sessions 2-5	Sessions 6-10	Sessions 11-15	Sessions 16-20	*Total*	Sessions 2-5	Sessions 6-10	Sessions 11-15	Sessions 16-20
Frequency of experience side effects(Mean (SD))										
Headache	13.25 (16.08)	2.75 (1.44)	3.88 (1.41)	3.50 (1.97)	3.13 (1.78)	8.25 (6.96)	2.56 (1.50)	2.50 (2.25)	1.69 (2.02)	1.50 (2.00)
Discomfort on head	9.19 (8.07)	2.00 (1.55)	2.50 (2.31)	2.56 (2.39)	2.13 (2.22)	5.44 (7.24)	2.00 (1.71)	1.44 (2.22)	1.06 (1.98)	0.94 (2.02)
Nausea	8.19 (7.48)	1.75 (1.65)	2.31 (2.33)	2.31 (2.12)	1.81 (1.94)	7.31 (7.65)	2.19 (7.65)	2.06 (1.38)	1.63 (2.36)	1.44 (2.25)
Dizziness	7.44 (7.62)	1.63 (1.75)	2.13 (2.31)	1.88 (2.06)	1.81 (2.07)	5.50 (7.13)	1.75 (1.73)	1.56 (2.10)	1.25 (1.95)	0.94 (2.02)
Light-headedness	8.13 (7.54)	1.81 (1.72)	2.13 (2.19)	2.31 (2.18)	1.88 (2.06)	6.69 (7.42)	2.00 (1.71)	1.88 (2.25)	1.50 (2.00)	1.31 (2.02)
Skin irritation	5.75 (6.82)	1.25 (1.39)	1.31 (1.74)	1.63 (2.16)	1.56 (1.97)	4.63 (7.40)	1.25 (1.73)	1.50 (2.31)	0.96 (1.88)	0.94 (2.02)
Drowsiness	10.38 (7.56)	2.25 (1.57)	3.00 (2.16)	2.88 (2.22)	2.25 (2.18)	7.06 (7.09)	1.88 (1.71)	2.38 (2.13)	1.38 (2.09)	1.44 (2.03)
Maximum intensity / severity of experienced side effects (Mean (SD))										
Headache	4.93 (2.29)	3.01 (2.69)	3.86 (2.33)	2.04 (2.06)	1.99 (2.42)	5.16 (2.77)	4.7 (3.05)	3.22 (3.00)	1.52 (1.83)	1.16 (1.84)
Discomfort on head	3.22 (3.26)	2.78 (3.11)	1.95 (3.07)	0.73 (0.96)	0.77 (1.62)	2.93 (3.14)	3.05 (3.21)	0.69 (1.80)	0.13 (0.29)	0.05 (0.16)
Nausea	2.91 (3.18)	2.34 (2.81)	1.67 (2.91)	0.58 (0.79)	0.91 (2.11)	3.46 (3.11)	2.42 (2.73)	1.34 (2.70)	1.46 (2.81)	1.09 (2.31)
Dizziness	1.70 (2.16)	1.08 (1.98)	0.99 (1.92)	0.56 (1.63)	0.91 (1.86)	3.29 (2.96)	2.05 (3.04)	1.05 (1.63)	1.31 (1.98)	0.19 (0.49)
Light-headedness	2.33 (2.60)	1.53 (2.15)	1.54 (2.44)	1.11 (2.09)	0.73 (1.73)	2.95 (3.01)	2.13 (2.97)	1.29 (2.18)	1.76 (2.69)	0.42 (0.75)
Skin irritation	0.81 (1.15)	0.39 (0.59)	0.45 (0.83)	0.34 (0.89)	0.33 (0.89)	0.68 (2.05)	0.44 (1.25)	0.67 (2.13)	0.19 (0.57)	0.05 (0.13)
Drowsiness	5.23 (3.60)	4.06 (3.41)	3.94 (3.62)	2.38 (2.80)	2.05 (2.58)	3.51 (3.16)	2.94 (3.28)	2.89 (2.74)	1.27 (2.59)	1.26 (2.14)

Supplementary Table 1. Mean frequency and mean maximum intensity / severity of experienced side effects.

SD = standard deviation

For peer review only

For peer review only - http://bmjopen.bmj.com/site/about/guidelines.xhtml

CONSORT 2010 checklist of information to include when reporting a pilot or feasibility trial*

Section/Topic	Item No	Checklist item	Reported on page No
**Title and abstract**			
	1a	Identification as a pilot or feasibility randomised trial in the title	1
	1b	Structured summary of pilot trial design, methods, results, and conclusions (for specific guidance see CONSORT abstract extension for pilot trials)	2
**Introduction**			
Background and objectives	2a	Scientific background and explanation of rationale for future definitive trial, and reasons for randomised pilot trial	3
	2b	Specific objectives or research questions for pilot trial	3
**Methods**			
Trial design	3a	Description of pilot trial design (such as parallel, factorial) including allocation ratio	4
	3b	Important changes to methods after pilot trial commencement (such as eligibility criteria), with reasons	6
Participants	4a	Eligibility criteria for participants	4
	4b	Settings and locations where the data were collected	4
	4c	How participants were identified and consented	4
Interventions	5	The interventions for each group with sufficient details to allow replication, including how and when they were actually administered	4
Outcomes	6a	Completely defined prespecified assessments or measurements to address each pilot trial objective specified in 2b, including how and when they were assessed	5
	6b	Any changes to pilot trial assessments or measurements after the pilot trial commenced, with reasons	N/A
	6c	If applicable, prespecified criteria used to judge whether, or how, to proceed with future definitive trial	5
Sample size	7a	Rationale for numbers in the pilot trial	6
	7b	When applicable, explanation of any interim analyses and stopping guidelines	6
Randomisation:			
Sequence generation	8a	Method used to generate the random allocation sequence	4
	8b	Type of randomisation(s); details of any restriction (such as blocking and block size)	4
Allocation concealment mechanism	9	Mechanism used to implement the random allocation sequence (such as sequentially numbered containers), describing any steps taken to conceal the sequence until interventions were assigned	Supplementary file, page 1

For peer review only

Implementation	10	Who generated the random allocation sequence, who enrolled participants, and who assigned participants to interventions	4 and Supplementary file, page 1
Blinding	11a	If done, who was blinded after assignment to interventions (for example, participants, care providers, those assessing outcomes) and how	4
	11b	If relevant, description of the similarity of interventions	5
Statistical methods	12	Methods used to address each pilot trial objective whether qualitative or quantitative	6
**Results**			
Participant flow (a diagram is strongly recommended)	13a	For each group, the numbers of participants who were approached and/or assessed for eligibility, randomly assigned, received intended treatment, and were assessed for each objective	6 and Figure 1
	13b	For each group, losses and exclusions after randomisation, together with reasons	6 and Figure 1
Recruitment	14a	Dates defining the periods of recruitment and follow-up	6
	14b	Why the pilot trial ended or was stopped	6
Baseline data	15	A table showing baseline demographic and clinical characteristics for each group	7
Numbers analysed	16	For each objective, number of participants (denominator) included in each analysis. If relevant, these numbers should be by randomised group	7
Outcomes and estimation	17	For each objective, results including expressions of uncertainty (such as 95% confidence interval) for any estimates. If relevant, these results should be by randomised group	8/9
Ancillary analyses	18	Results of any other analyses performed that could be used to inform the future definitive trial	8
Harms	19	All important harms or unintended effects in each group (for specific guidance see CONSORT for harms)	11
	19a	If relevant, other important unintended consequences	N/A
**Discussion**			
Limitations	20	Pilot trial limitations, addressing sources of potential bias and remaining uncertainty about feasibility	12
Generalisability	21	Generalisability (applicability) of pilot trial methods and findings to future definitive trial and other studies	12
Interpretation	22	Interpretation consistent with pilot trial objectives and findings, balancing potential benefits and harms, and considering other relevant evidence	11
	22a	Implications for progression from pilot to future definitive trial, including any proposed amendments	12
**Other information**			
Registration	23	Registration number for pilot trial and name of trial registry	14
Protocol	24	Where the pilot trial protocol can be accessed, if available	14
Funding	25	Sources of funding and other support (such as supply of drugs), role of funders	14

For peer review only

	26	Ethical approval or approval by research review committee, confirmed with reference number	4

Citation: Eldridge SM, Chan CL, Campbell MJ, Bond CM, Hopewell S, Thabane L, et al. CONSORT 2010 statement: extension to randomised pilot and feasibility trials. BMJ. 2016;355.

*We strongly recommend reading this statement in conjunction with the CONSORT 2010, extension to randomised pilot and feasibility trials, Explanation and Elaboration for important clarifications on all the items. If relevant, we also recommend reading CONSORT extensions for cluster randomised trials, non-inferiority and equivalence trials, non-pharmacological treatments, herbal interventions, and pragmatic trials. Additional extensions are forthcoming: for those and for up to date references relevant to this checklist, see www.consort-statement.org.

For peer review only
